# Crawling and Gliding: A Computational Model for Shape-Driven Cell Migration

**DOI:** 10.1371/journal.pcbi.1004280

**Published:** 2015-10-21

**Authors:** Ioana Niculescu, Johannes Textor, Rob J. de Boer

**Affiliations:** Theoretical Biology & Bioinformatics, Utrecht University, The Netherlands; University of British Columbia, CANADA

## Abstract

Cell migration is a complex process involving many intracellular and extracellular factors, with different cell types adopting sometimes strikingly different morphologies. Modeling realistically behaving cells in tissues is computationally challenging because it implies dealing with multiple levels of complexity. We extend the Cellular Potts Model with an actin-inspired feedback mechanism that allows small stochastic cell rufflings to expand to cell protrusions. This simple phenomenological model produces realistically crawling and deforming amoeboid cells, and gliding half-moon shaped keratocyte-like cells. Both cell types can migrate randomly or follow directional cues. They can squeeze in between other cells in densely populated environments or migrate collectively. The model is computationally light, which allows the study of large, dense and heterogeneous tissues containing cells with realistic shapes and migratory properties.

## Author Summary

Cell migration is involved in vital processes like morphogenesis, regeneration and immune system responses, but can also play a central role in pathological processes like metastasization. Computational models have been successfully employed to explain how single cells migrate, and to study how diverse cell-cell interactions contribute to tissue level behavior. However, there are few models that implement realistic cell shapes in multicellular simulations. The method we present here is able to reproduce two different types of motile cells—amoeboid and keratocyte-like cells. Amoeboid cells are highly motile and deform frequently; many cells can act amoeboid in certain circumstances e.g., immune system cells, epithelial cells, individually migrating cancer cells. Keratocytes are (fish) epithelial cells which are famous for their ability to preserve their shape and direction when migrating individually; during wound healing, keratocytes migrate collectively, in sheets, to the site needing reepithelialization. Our method is computationally simple, improves the realism of multicellular simulations and can help assess the tissue level impact of specific cell shapes. For example, it can be employed to study the tissue scanning strategies of leukocytes, the circumstances in which cancer cells adopt amoeboid migration strategies, or the collective migration of keratocytes.

This is a *PLOS Computational Biology* Methods paper.

## Introduction

Single migrating cells fall into different morphological categories [1]. For example, keratocyte-like cells, named after fish epithelial cells, retain a half-moon shape and glide quite persistently, led by a lasting, thin and broad lammellipodium [2, 3]. In contrast, amoeboid cells change their shape frequently and migrate erratically by extending and retracting protrusions [4]. Leukocytes are classical examples of amoeboid cells patrolling through dense tissue. For instance, T lymphocytes squeeze through the tightly packed epidermis to find and fight infections [5–7].

Theoretical models are useful tools for exploring the relation between cell shape, cell migration and their tissue level implications, because they allow experiments on virtual cells in controlled environments. Categorized by scale, cell-based models can be single-cell or multicellular. Single-cell models answer questions about cell behavior by implementing various intracellular mechanisms, while multicellular models answer questions at the tissue level by using simplified representations of individual cells in order to reduce computational complexity.

Several single-cell models explain the shape and behavior of individual cells biomechanically [2, 3, 8–12] or by other intracellular mechanisms [13, 14]. Although they vary greatly in their goal and level of detail, these models generally obtain migration by using a variant of the “local activation global inhibition” mechanism [15–18], and they usually require many equations and parameters in order to impose physical forces [2, 3], to allow forces to emerge [8], and/or to diffuse intracellular molecules [13, 14].

Multicellular models typically use simplified cell shapes—points, spheroids, collections of lattice sites—and impose cell migration by means of vectors or other directional signals. The few multicellular models that combine cell shapes with the associated migration patterns demonstrated that realistic cell representations can be essential for the behavior that emerges at the tissue level [7, 19, 20].

In general, single-cell models that result in realistic shape and migration are computationally expensive and therefore difficult to use in a multicellular context. Multicellular models, on the other hand, tend to strip the cells of their inherent shape-migration interconnection. We propose a simple and computationally light phenomenological model of cell migration, implemented within the Cellular Potts Model (CPM), that faithfully reproduces cell shapes and their associated migration properties, and can readily be used in big multicellular simulations. The cells in our model reproduce amoeboid and keratocyte behavior, migrate randomly with different levels of persistence, and display qualitative traits of chemotaxing cells. We illustrate the capabilities of our model in a multicellular context with 2 experiments: one experiment with T lymphocytes migrating in tightly packed skin, and one experiment with keratocytes migrating collectively. We show that our mechanism merely increases the computation time of the CPM by a small percentage, and that this percentage stays constant with increasing cell numbers, making the mechanism scalable and therefore suited for large simulations.

## Results and Discussion

In this section we present the Act model, discuss its properties and we show that it reproduces realistically looking and behaving cells. We demonstrate its use in multicellular contexts by applying it in two different multicellular systems and showing that it adds little computational load to the basic CPM.

### The Act model

Our new computational model, the Act model, is inspired by actin dynamics. It extends the CPM with a local feedback mechanism resulting in cell protrusions and, as a consequence, in cell motility. The mechanism amplifies the inherent membrane fluctuations of CPM cells in a manner depending on the size of the fluctuations and their recent protrusive activity.

The protrusive activity is tracked by keeping an activity value for every lattice site. The empty lattice sites that form the medium have a zero activity value, while sites that are freshly incorporated by a cell get the maximum activity value (Max_Act_). The activity value of a site decreases by one after every MCS, until it reaches zero, creating a memory of Max_Act_ MCSs in which the site “remembers” that it was active. The combination of the memory of a site *u* with the activity in its neighborhood forms the basis for a local positive feedback mechanism that biases the copy attempt from the active site *u* to a less active site *v*.

**Fig 1:**
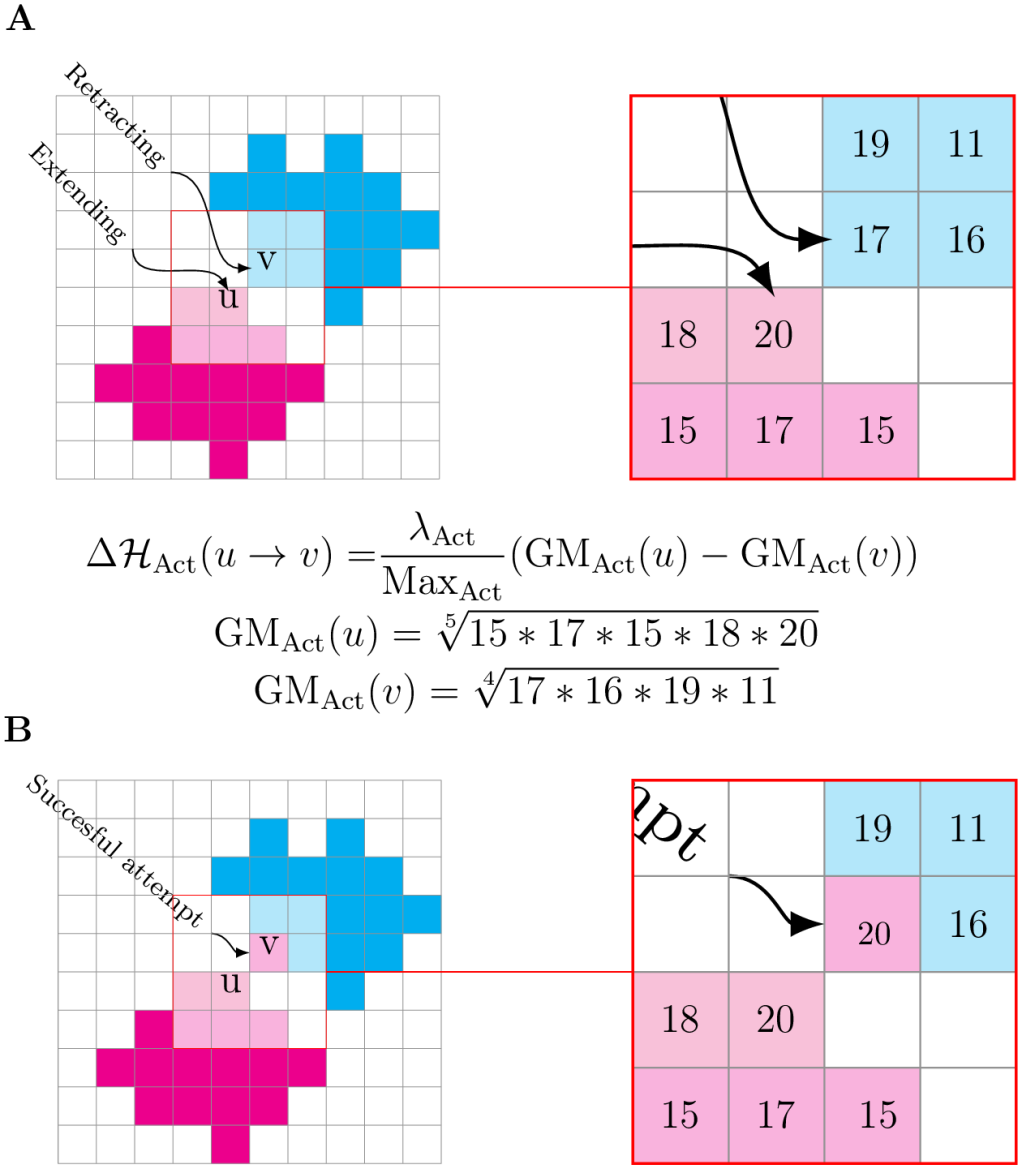
Example of a CPM copy attempt with the Act model. In the Act model, we encourage copying from sites with relatively high activity values to sites with lower values. (A) The magenta cell is attempting to copy itself into the cyan cell by extending from lattice site *u* into the lattice site *v*. The right inset magnifies the intracellular neighborhoods of *u* (light magenta) and *v* (light cyan); the lattice sites contain examples of activity values. We obtain GM_Act_(*u*) and GM_Act_(*v*) by geometrically averaging over the activity values of each neighborhood, and we calculate $\Delta H_{\text{Act}}$ from the difference of these values. The success probability of the copy attempt is biased by subtracting $\Delta H_{\text{Act}}$ from ΔH. In this example (i.e., Max_Act_ = 20, *λ*_Act_ > 0), $\Delta H_{\text{Act}}\left(u\to v\right)=\lambda_{\text{Act}}\frac{1.46}{20}>0$ which increases the chance of accepting the copy attempt. (B) If the copy attempt is successful, *v* is incorporated into the magenta cell and the site is assigned the maximum activity value (in this case, Max_Act_ = 20).

Formally, the mechanism is implemented by subtracting the term $\Delta H_{\text{Act}}\left(u\to v\right)=\lambda_{\text{Act}}/{\text{Max}}_{\text{Act}}({\text{GM}}_{\text{Act}}(u)-{\text{GM}}_{\text{Act}}(v))$ from the energy difference of the system, ΔH. The parameter *λ*_Act_ is the maximum contribution of the Act model to the Hamiltonian. The average activity around site *u* is calculated as the geometric mean of the activity values in the neighborhood of *u*, ${\text{GM}}_{\text{Act}}\left(u\right)={\left(\prod_{\;y\in V(u)}\text{Act}\left(y\right)\right)}^{1/|V(u)|}$, with *V*(*u*) the direct Moore neighborhood of *u* that belongs to the same cell as *u* and 0 ≤ GM_Act_(*u*) ≤ Max_Act_. Compared to the arithmetic mean, the geometric mean favors the contribution to the Hamiltonian of complete Moore neighborhoods with consistently high activity values; it diminishes the contribution of Moore neighborhoods with low activity values and it nullifies the contribution of Moore neighborhoods “with holes” (i.e., lattice sites with activity value zero). The dynamics of a copy trial are shown in Fig 1.

$\Delta {ℋ}_{Act}$ can be interpreted as the force resulting from pushing and resistance at the membrane element between *u* and *v*. This force reaches a maximum when *u*, backed up by a recent active neighborhood (GM_Act_(*u*) = Max_Act_) tries to extend into a lattice site that opposes no resistance derived from activity (GM_Act_(*v*) = 0).

The Act model encourages relatively big patches of active lattice sites just behind the cell membrane to extend further, creating protrusions in a way that roughly resembles protrusion formation in real cells, where branched networks of actin filaments grow behind the membrane and push it outwards. Within an actin network, the actin globular subunits attach at the barbed ends of the filaments, age and detach some distance behind the leading edge [21]. In our model, newly incorporated lattice sites also “age” and “detach” as their activity values decay until they reach zero and the sites cease to be part of the active patch. From this perspective, the Max_Act_ parameter can be interpreted as the expected lifetime of an actin globular subunit within an actin network and the *λ*_Act_ parameter can be seen as the maximum protrusive force of the network. The parallelism between actin globular subunits and active lattice sites motivates also our choice for the geometric average instead of the arithmetic average in the calculation of GM_Act_(*u*). New actin subunits contribute to actin network growth by attaching to the existing network structure, therefore, in our model, new active sites need to adjoin the active patch they originate from. Because the arithmetic mean disperses the activity values, it potentially creates active sites that are disconnected from active patches, while the geometric mean prevents this effect.

#### The cell breaks symmetry, polarizes and starts moving

In order to migrate, eukariotic cells first need to break symmetry—which can happen randomly [22], or due to directional cues [23]—and subsequently polarize. In the Act model, a cell breaks symmetry spontaneously and is considered to be polarized when it presents one clearly visible leading edge.

**Fig 2:**
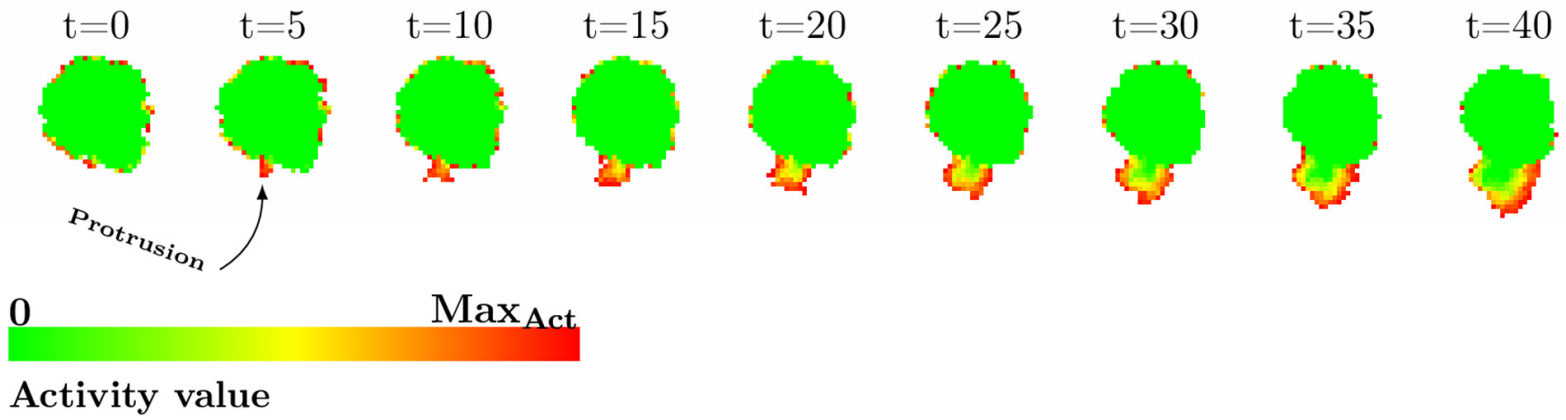
Random breaking of symmetry results in polarization. Time series of the initiation of a protrusion within the Act model. At time t = 0 MCSs, the cell asserts random membrane fluctuations with no clear bias towards any direction. At t = 5 MCSs, a small protrusion starts forming at the bottom of the cell. Due to a consistently active neighborhood at the protrusion, the positive feedback is encouraging it to extend further. Within the protrusion a gradient of activity values develops resembling the treadmilling of the actin network [21].

Symmetry breaking starts with several stochastic membrane rufflings that occur more or less simultaneously next to each other on the cell membrane. If the rufflings happen to accumulate high activity values and form a large enough active patch, they will have a higher likelihood to extend and thereby create new elements with maximum activity values. This leads to a positive feedback where a patch of increased activity is encouraged to grow. Ultimately this microscopic process forms a macroscopic protrusion (Fig 2).

The local positive feedback mechanism is counterbalanced by the global inhibition mechanism emerging from the cell area and perimeter constraints (see Methods), which limit how much the cell can extend. As a consequence, once a sufficiently large protrusion has formed, other protrusions become less probable and the cell becomes polarized. If the polarized cell extends its leading protrusion even further, the rear of the cell will retract causing the cell to change its position on the lattice.

In conclusion, the Act model reproduces phenomenologically an important behavior of real cells: random actin-based breaking of symmetry. The positive feedback that forms the basis of the Act model could, in real cells, be driven by a preferential localization of Arp2/3 or other actin-associated proteins at sites of recent actin polymerization [24–26]. Inside a growing protrusion, the activity values of individual sites form a spatial pattern resembling actin network treadmilling [21]: the lattice sites are youngest at the protruding membrane and increasingly older the further they are located from the leading edge of the protrusion (Fig 2 and S1 Video).

#### The Act model reproduces amoeboid and keratocyte behavior

We explored the Act model through simulations with different parameters and discovered that it reproduces two contrasting types of migration: amoeboid and keratocyte-like.

**Fig 3:**
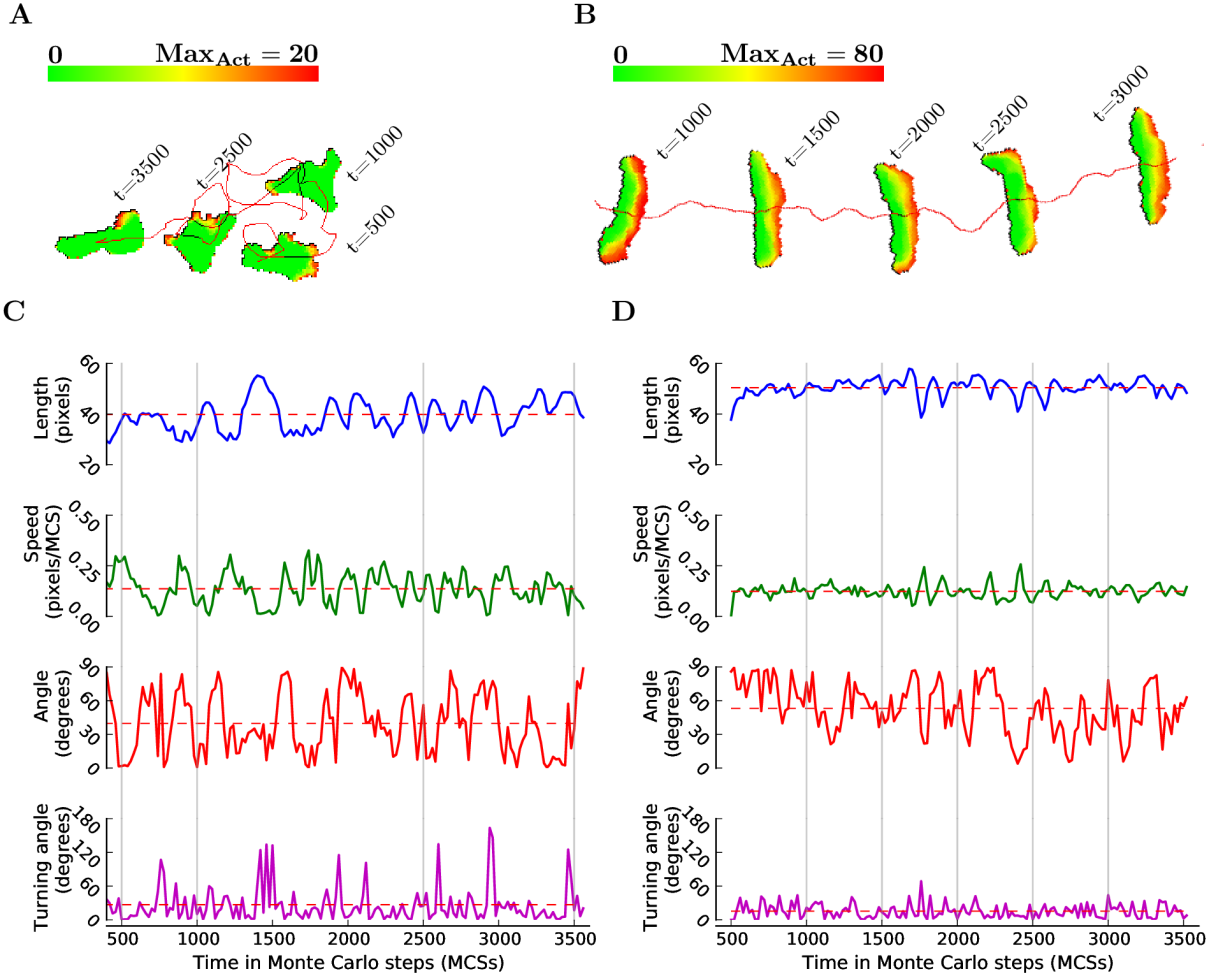
The Act model reproduces amoeboid and keratocyte-like behavior. (A) Typical appearance and migration behavior of an amoeboid cell; cell track in red. (B) Typical appearance and migration behavior of a keratocyte-like cell. The colorbars represent the activity values scaled from 0 (green) to Max_Act_ (red). (C) Traces of the instantaneous migration features corresponding to the amoeboid cell in (A). (D) Traces of the instantaneous migration features corresponding to the keratocyte-like cell in (B). Vertical gray lines highlight the time points at which the cell snapshots were taken. Red dashed lines show the average values of the migration features calculated over the whole track. See Methods section for definitions of measurements and for the complete list of parameter values.

The amoeboid cell (Fig 3A and 3C) deforms frequently and has an erratic track, moving with high fluctuations in length, speed and turning angle (Fig 3A and 3C). The orientation-direction angle is fairly small, around 35 degrees on average, which suggests that the cell tends to move more often than not along its longest axis (Fig 3C).

Conversely, the keratocyte-like cell (Fig 3B and 3D) preserves an elongated, half moon shape and moves along a fairly straight track with almost constant length, speed and turning angle (Fig 3B and 3D). The orientation-direction angle is large, with an average close to 55 degrees, implying that the cell tends to move more often than not perpendicular to its longest axis (Fig 3D).

**Fig 4:**
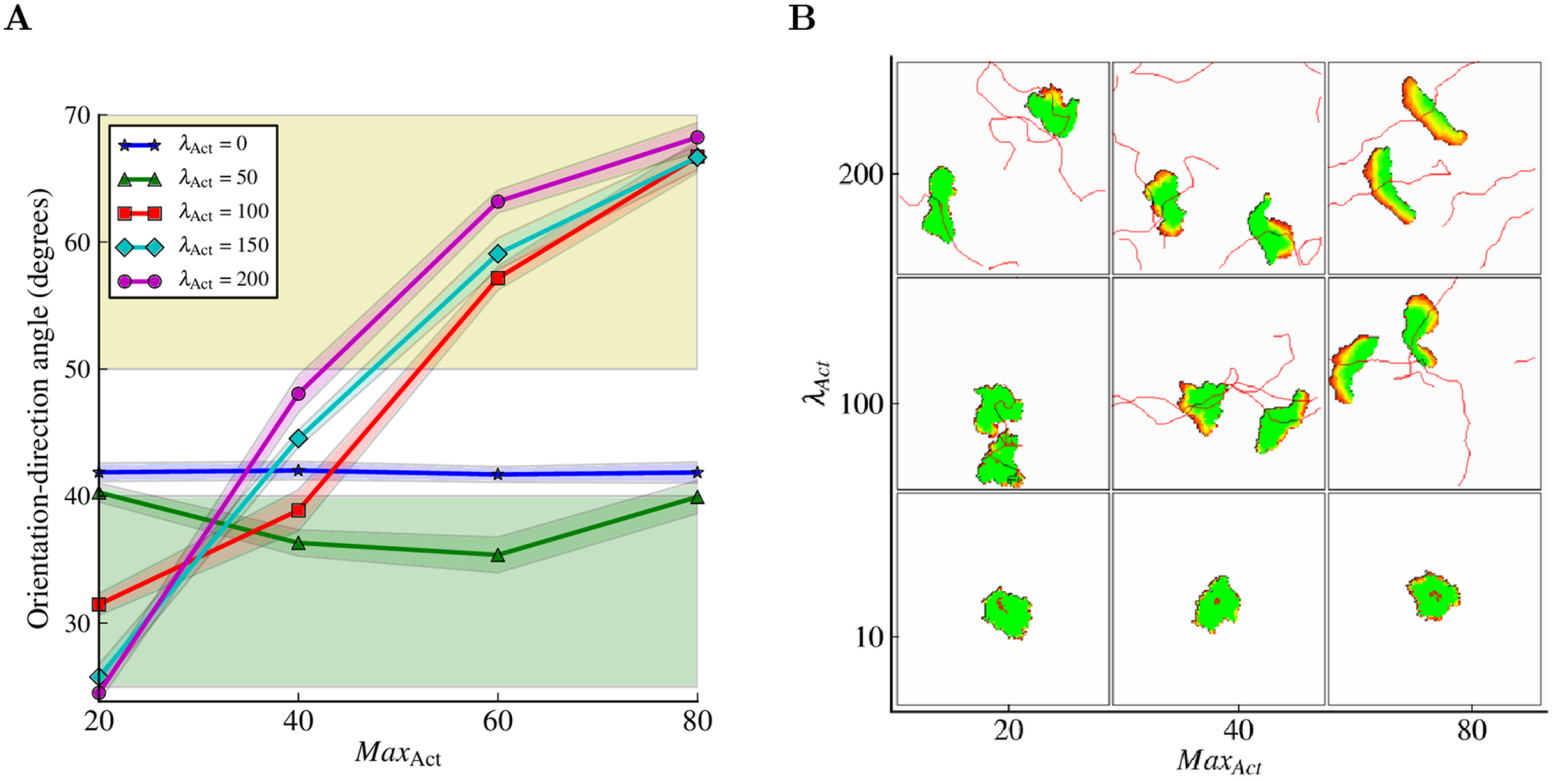
Amoeboid to keratocyte-like transition. (A) Low orientation-migration angles are typical for amoeboid cells (green region); high ones indicate transversal migration which is typical for the keratocyte-like cells (yellow region). For large enough *λ*_Act_, there is a transition between amoeboid behavior at low Max_Act_ values and keratocyte-like behavior at high Max_Act_ values. *λ*_Act_ amplifies the amoeboid or keratocyte-like behavior, but cannot trigger a switch from one type of behavior to another on its own (e.g., no *λ*_Act_ value combined with a low Max_Act_ will result in keratocyte-like behavior). Every point in the graph represents the mean of 10 experiments of 30.000 MCSs each with sampling time Δ*t* = 20 MCSs between consecutive measurements. The shadows represent the standard deviations. (B) Morphospace of the Act model illustrating cell behavior at different combinations of parameter values. Every cell is showed at two positions along its track, except for the non-migrating cells. At *λ*_Act_ = 10 the cells are roundish and stationary: very similar to the basic CPM cells. At high *λ*_Act_ values and low Max_Act_ values the cells are amoeboid; at high *λ*_Act_ values and high Max_Act_ values the cells are keratocyte-like (see also S2 Video). See Methods section for definitions of measurements and for the complete list of parameter values.

**Fig 5:**
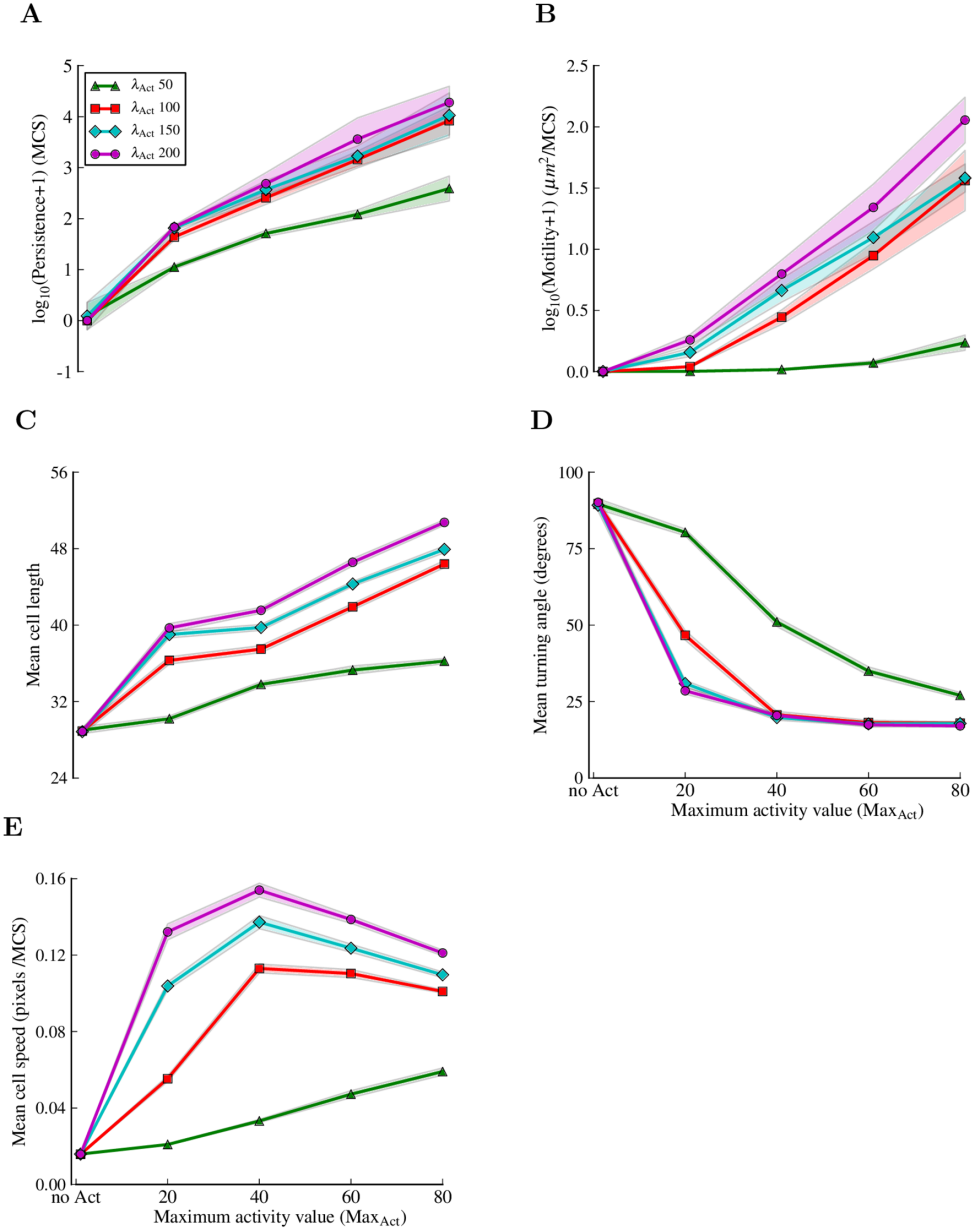
Migration features of cells in the Act model. Increasing the Max_Act_ and *λ*_Act_ parameters makes cells (A) more persistent, (B) more motile, and (C) longer. (D) The average turning angle of the cells decreases with both Max_Act_ and *λ*_Act_ from the random 90 degrees (typical for the jiggling of the basic CPM cells) to average values as low as 20 degrees as the cells migrate more organized. (E) The instantaneous speed of cells initially increases with Max_Act_ and *λ*_Act_, then decreases. The first point on the x axis represents the original CPM setting, without the Act model, i.e *λ*_Act_ = 0, Max_Act_ = undefined. The migration features were extracted from single cell experiments. Every point in a graph represents the mean of 10 simulations of 30.000 MCSs each with sampling time Δ*t* = 20 MCSs between consecutive measurements. The shadows represent the standard deviation. See Methods section for definitions of measurements and for the complete list of parameter values.

The orientation-direction angle conveniently discriminates between amoeboid and keratocyte-like cells; angles smaller than 40 degrees indicate migration in the direction of orientation which tends to be more amoeboid, while angles larger than 50 degrees indicate a more transversal, keratocyte-like type of migration (Fig 4A). When sweeping through the two parameters defining the Act model (i.e., Max_Act_ and *λ*_Act_), the orientation-direction angle shows a smooth transition from amoeboid to keratocyte-like behavior along the Max_Act_ axis (Fig 4A). This transition is confirmed by the “morphospace” of cells for different values of the Max_Act_ and *λ*_Act_ parameters (Fig 4B). Consistently, other migration features also shift from amoeboid to keratocyte-like as Max_Act_ increases: the persistence, motility and cell length increase (Fig 5A–5C), while the turning angle decreases (Fig 5D). The instantaneous cell speed grows with increasing Max_Act_ for amoeboid cells (i.e., orientation-direction angle ≥ 40) and decreases with increasing Max_Act_ for the keratocyte-like cells (i.e., orientation-direction angle ≤ 50; Fig 5E). This seemingly contradictory result—keratocyte-like cells are more motile than amoeboids (Fig 5B), but move slower—is a consequence of how speed is typically measured (see Methods). Frequent deformations make the centroids of amoeboid cells jump and jiggle, while the centroids of keratocyte-like cells displace in a smoother manner.

Interestingly, Max_Act_ is the only parameter responsible for the switch between amoeboid and keratocyte-like behavior. It defines the interval (in MCSs) over which a site remains active (memory) and causes a spatial effect proportional to its value (compare the active areas of cells in Fig 3). A small Max_Act_ value causes cell sites to “forget” they were active after short periods of inactivity, resulting in thin areas of activity behind cell protrusions, which make the protrusions more prone to disruptions or deletions by random membrane retractions. This leads to amoeboid migration with frequent changes in direction due to frequent protrusion extensions, splittings and retractions (see S3 Video). A high Max_Act_ value causes cell sites to “remember” they were active even after longer periods of inactivity, and creates thick activity areas behind protrusions which eventually merge to form a wide leading edge which is resilient to random membrane retractions. This leads to keratocyte-like migration with high persistence and stable cell shape (see S4 Video).

To summarize, the value of Max_Act_—which is directly related to the strength of positive feedback that amplifies the cell membrane fluctuations—correlates with a transition from amoeboid to keratocyte behavior. This implies that, in our model, increasing the positive feedback allows cells to switch from amoeboid to keratocyte-like migration.

#### Chemotaxis

**Fig 6:**
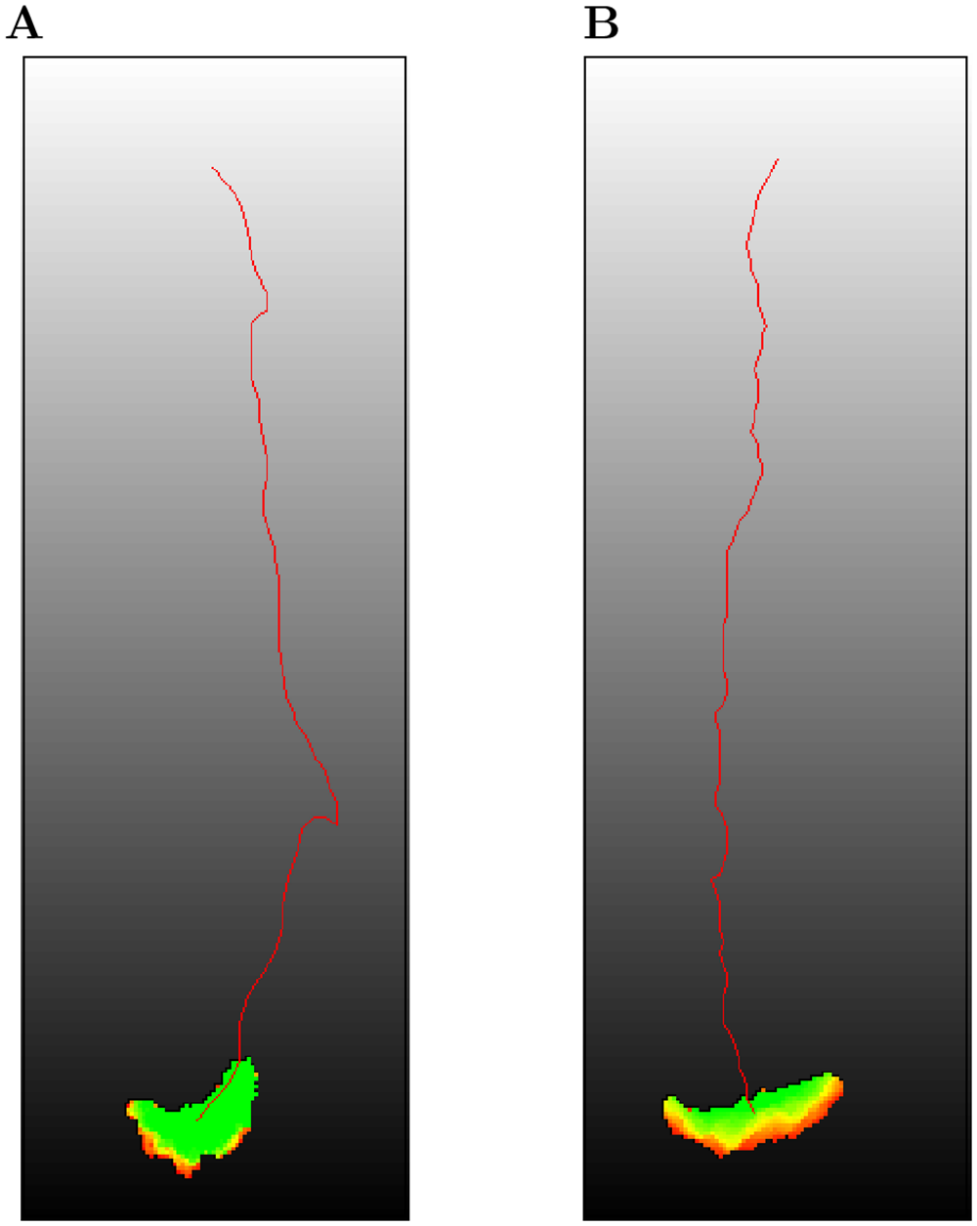
Amoeboid and keratocyte chemotaxis. (A) Amoeboid cells (*λ*_Act_ = 200, Max_Act_ = 20) and (B) keratocyte-like cells (*λ*_Act_ = 200, Max_Act_ = 80) were placed in linear chemokine gradients (slope 0.33, *λ*_Chemotaxis_ = 150) along the y axis (white to black gradient). Cell tracks are colored in red. See also the corresponding movies S5 and S6 Videos.

The ability of a cell to move along a chemical gradient is an essential process that has been extensively explored theoretically [22]. Most mathematical models focus on explaining chemokine gradient sensing and cell polarization [22], with just a few considering actual cell movement [13, 27–30]. We next show that the Act model, combined with the existing CPM method for modeling chemotaxis, results in cells moving up a chemokine gradient, while reproducing several other qualitative traits of real cells performing chemotaxis (Fig 6, S5 and S6 Videos).

**Fig 7:**
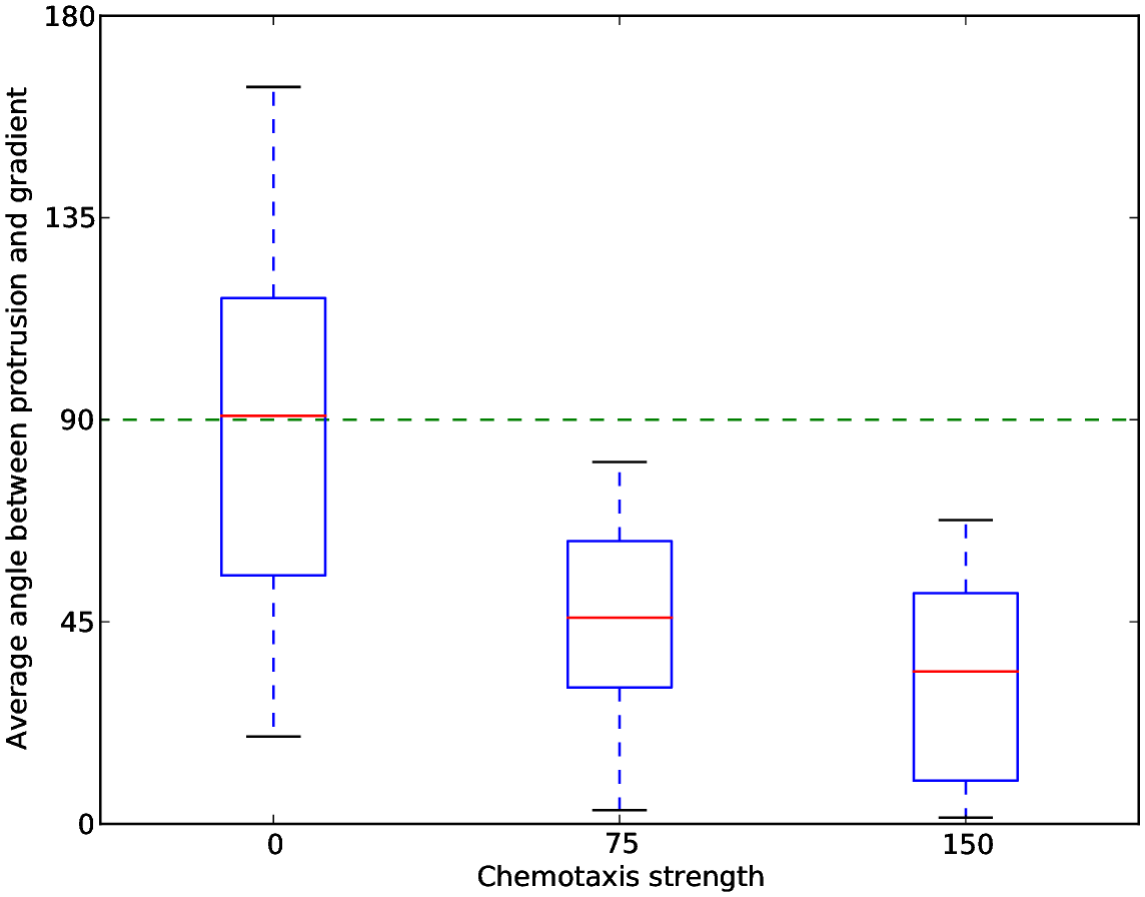
The direction of cell protrusions is biased by the chemotaxis strength. Amoeboid cells (*λ*_Act_,Max_Act_ = 20) were placed in chemokine gradients at different values of the chemotaxis strength (*λ*_Chemotaxis_). For every value of *λ*_Chemotaxis_, we conducted 25 independent simulations resulting in 25 images of single cells on which we performed image segmentation to extract the protrusions. The number of protrusions and the angles they made with the chemokine gradient were averaged over the 25 experiments. The average protrusion-gradient angle decreases as *λ*_Chemotaxis_ increases, while the average number of simultaneous cell protrusions remains constant (1.6 protrusions).

The CPM method for chemotaxis used for our model (see Methods) encourages cell membrane segments to extend up the gradient, creating active patches. These patches can trigger the positive feedback mechanism of the Act model, resulting in cells extending protrusions up the gradient with a higher frequency than in other directions (Fig 7). This is consistent with the behavior of real amoeboid cells which produce more pseudopodia at the side of the cell that is exposed to the highest chemoattractant level [31].

**Fig 8:**
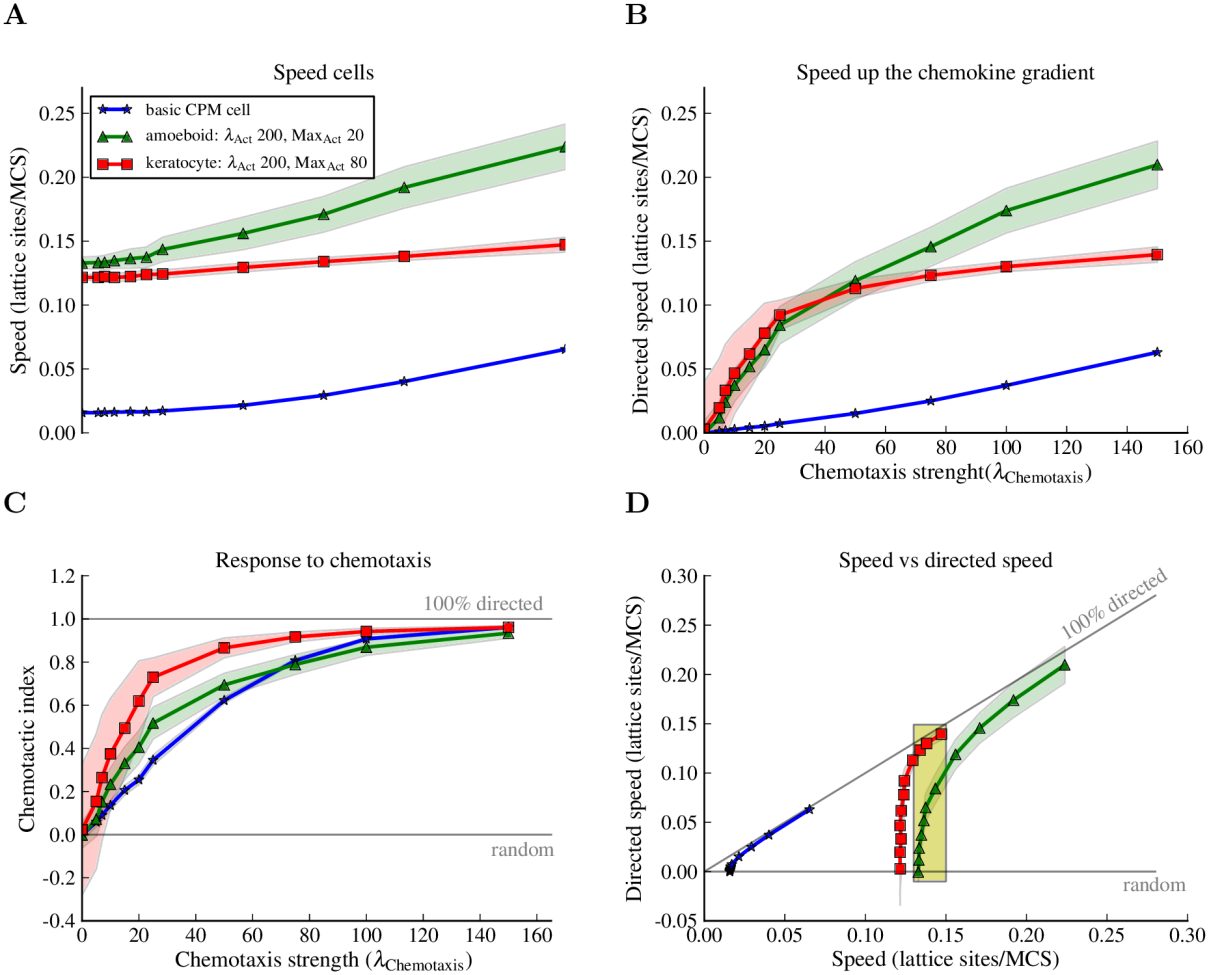
The Act model makes cells more sensitive to chemotaxis. Basic CPM cells (*λ*_Act_ = 0), amoeboid cells (*λ*_Act_ = 200, Max_Act_ = 20) and keratocyte-like cells (*λ*_Act_ = 200, Max_Act_ = 80) were placed in chemotactic gradients. The average instantaneous speed and the average directed speed were measured for different values of the chemotaxis strength parameter (*λ*_Chemotaxis_). Every point in a graph is the average of 300 single cell simulations of 5000 MCSs each with sampling time Δ*t* = 20 MCSs between consecutive measurements. The blue, red and green shadows represent the standard deviations. (A) The speed of cells increases with increasing *λ*_Chemotaxis_ values; the speed of the amoeboid cell grows much faster than that of the keratocyte-like cells. (B) The speed up the chemokine gradient is growing with *λ*_Chemotaxis_. (C) The Act model makes keratocyte-like and amoeboid cells more sensitive to the chemokine gradient; they migrate more directionally than the basic CPM cells at low values of *λ*_Chemotaxis_. (D) The directed speed of the keratocyte is higher when cells have the same speed (yellow highlighted region). See Methods section for definitions of measurements and for the complete list of parameter values.

Because in the CPM the chemotaxis signal is implemented as a force that makes the cells move proportionally to the chemotactic strength parameter *λ*_Chemotaxis_ [32], the speed of basic CPM cells, amoeboid cells and keratocyte-like cells increases with *λ*_Chemotaxis_ (Fig 8A and 8B). To evaluate and compare the chemotactic response of different cell types moving with different average speeds (Fig 8A), we used the directed speed and the chemotactic index as measures of chemotactic “sensitivity”. The chemotactic sensitivity increases when increasing the value of the chemotactic strength parameter, *λ*_Chemotaxis_ (Fig 8B and 8C) because cells are increasingly constrained to extend protrusions (amoeboid cells) or to form leading edges (keratocyte-like cells) up the gradient. Increasing the value of *λ*_Chemotaxis_ in the CPM is equivalent to steepening the chemokine gradient (see Methods), hence the chemotactic sensitivity of our *in silico* cells correlates with the steepness of the chemokine gradient. This is similar to the response of *Dictyostelium discoideum* cells when placed in cAMP gradients of different steepness [33].

The keratocyte-like cells are most sensitive to the chemokine: they sense the gradient at low chemotaxis strength values and migrate more directionally than the other cells (Fig 8C). This is confirmed by a higher directed speed of the keratocyte-like cells in the small parameter region in which amoeboid and keratocyte-like cells have the same instantaneous speed (Fig 8D, yellow region).

To summarize, despite its qualitative and phenomenological nature, our model combined with chemotaxis gives rise to several realistic traits of amoeboid chemotactic migration. Because fish keratocytes are not known to respond efficiently to chemoattractants [34], it is not possible to qualitatively validate the chemotaxis of keratocyte-like cells.

#### Multicellular migration

Until this point, we have described the properties of the Act model at the level of single cells. Next we show the potential of the model in complex multicellular systems with two experiments: in the first one we reproduce and analyze the collective migration of keratocytes, and in the second one we explore the behavior of the Act cells when migrating in tissues with different properties. Szabo et al. [35] described with an *in vitro* experiment a density dependent steep phase transition in the collective migration of keratocytes. At low densities, keratocyte migration was uncoordinated, while at higher densities their migration became organized and collective. The authors reproduced those *in vitro* findings with a computational model that represents cells as particles exhibiting self-propulsion in the direction of their displacement.

**Fig 9:**
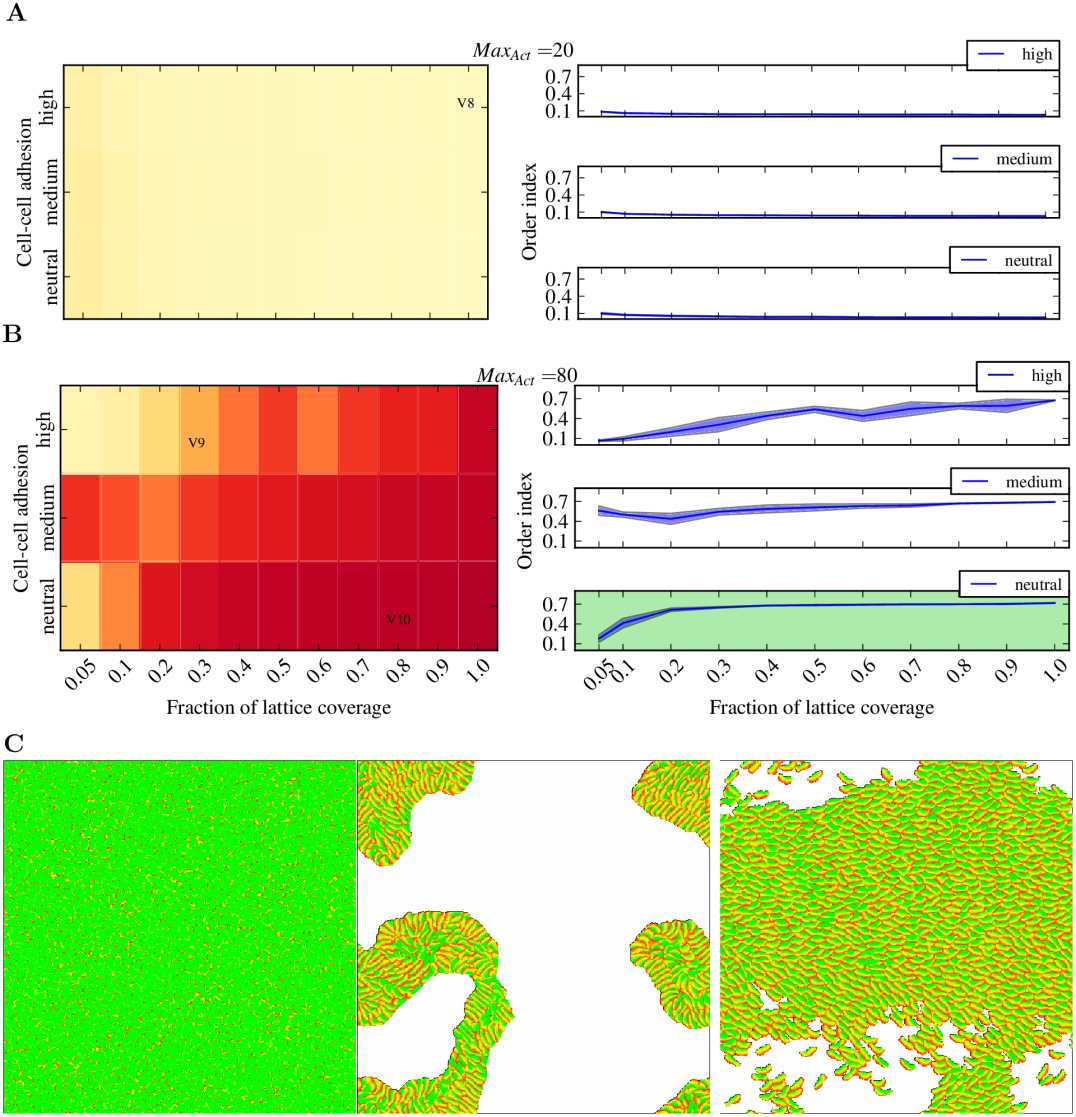
Collective migration of the Act cells. (A,B) Heatmaps (left column) and plots (right column) of the order index for Act cells with Max_Act_ = 20 (A) or Max_Act_ = 80 (B). The order index (see Methods) ranges from 0 (complete random migration) to 1 (completely collective migration). The color in the heatmaps ranges from yellow (order index 0) to red (order index 0.8). Act cells were placed on the lattice at different coverages and different cell-cell adhesion values, and, after a period of relaxation, the mean order index was calculated for every experiment (see Methods). Every tile in the heatmaps and every point in the plots represent the average of 30 simulations. The heatmaps are annotated with the names of the videos corresponding to the particular parameters set of the tile. (C) Representative snapshots of S8 Video (left), S9 Video (middle), and S10 Video (right). See Methods section for the complete list of parameter values.

Because of the way cells are represented, the Szabo model [35] does not allow to investigate whether collective migration depends on a particular cell type. We used the Act model to address this question because it realistically represents cell shape, and we used the standard CPM parameters to represent cell-cell adhesion. This allowed us to define the conditions needed for collective cell migration by an experiment in which we varied cell type (by varying Max_Act_), cell-cell adhesion (see Methods) and the fraction of lattice covered by cells. We found that amoeboid cells do not migrate collectively, regardless of cell-cell adhesion and lattice coverage (Fig 9A, S8 Video). Keratocyte-like cells do migrate collectively (Fig 9B). We found that simply placing keratocyte-like cells at different densities on the lattice reproduces qualitatively the *in vitro* behavior [35]: at high cell densities the cells migrate collectively, while at low cell densities the migration remains uncoordinated. Adhesion has a great impact on the phase transition between uncoordinated and collective migration: high cell-cell adhesion increasing the lattice coverage results in a linear increase in collectivity of the keratocyte-like cells (Fig 9B).

The keratocytes stick to each other and influence each other’s directions, which can result in the emergence of cell vortices (see S9 Video) that disturb the collective behavior of the population. Interestingly, we observed the experimentally reported steep transition from non-collective to collective migration [35] only at the neutral value for cell-cell adhesion (Fig 9B, plot with green background). In this case, the cells are able to gently push each other without the disrupting effects of high adhesion (see S10 Video).

**Fig 10:**
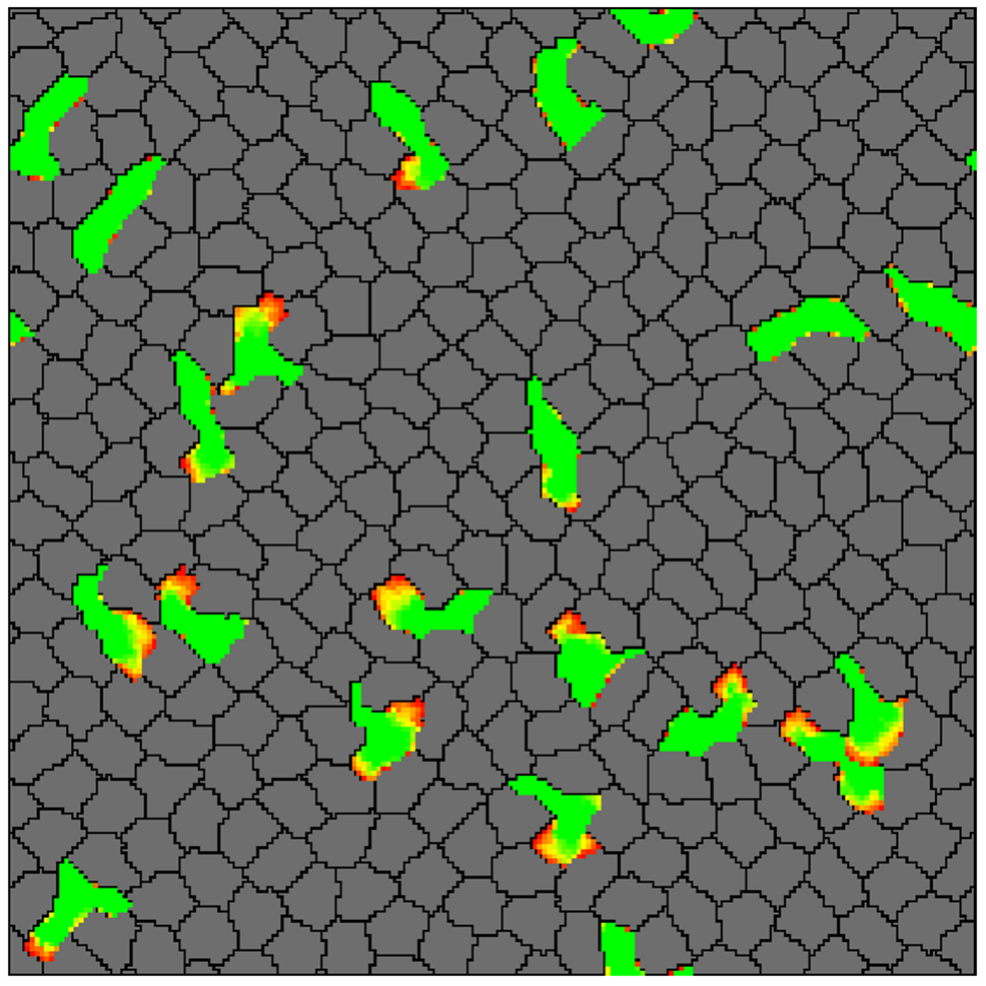
Effector T cells patrolling in the tightly packed environment of the epidermis. In silico skin cells (gray) and T cells (green) were seeded at 100% lattice coverage on a wrapped lattice. The T cells are extending protrusions (yellow to red gradient) and squeeze between skin cells by pushing them apart (see also S7 Video). See Methods section for the complete list of parameter values.

The second experiment is inspired by immunology, where, following infection or inflammation in the skin, amoeboid behaving leukocytes need to squeeze through and scan a tightly packed environment to locate tissue damage and/or the pathogen [5–7]. As proof of principle, we reproduced the epidermis environment *in silico* by placing stationary and almost rigid cells at a high density on our lattice, and we seeded amoeboid cells representing T cells between them. Although the Act model by itself only provides a feedback mechanism, and no persistence or polarity, the *in silico* T cells perform a persistent random walk, and are able to squeeze themselves through the dense *in silico* tissue in a manner resembling effector T cells crawling through the epidermis (compare Fig 10 and S7 Video to the Supplementary Movie 3 in Ariotti et al. [7]).

**Fig 11:**
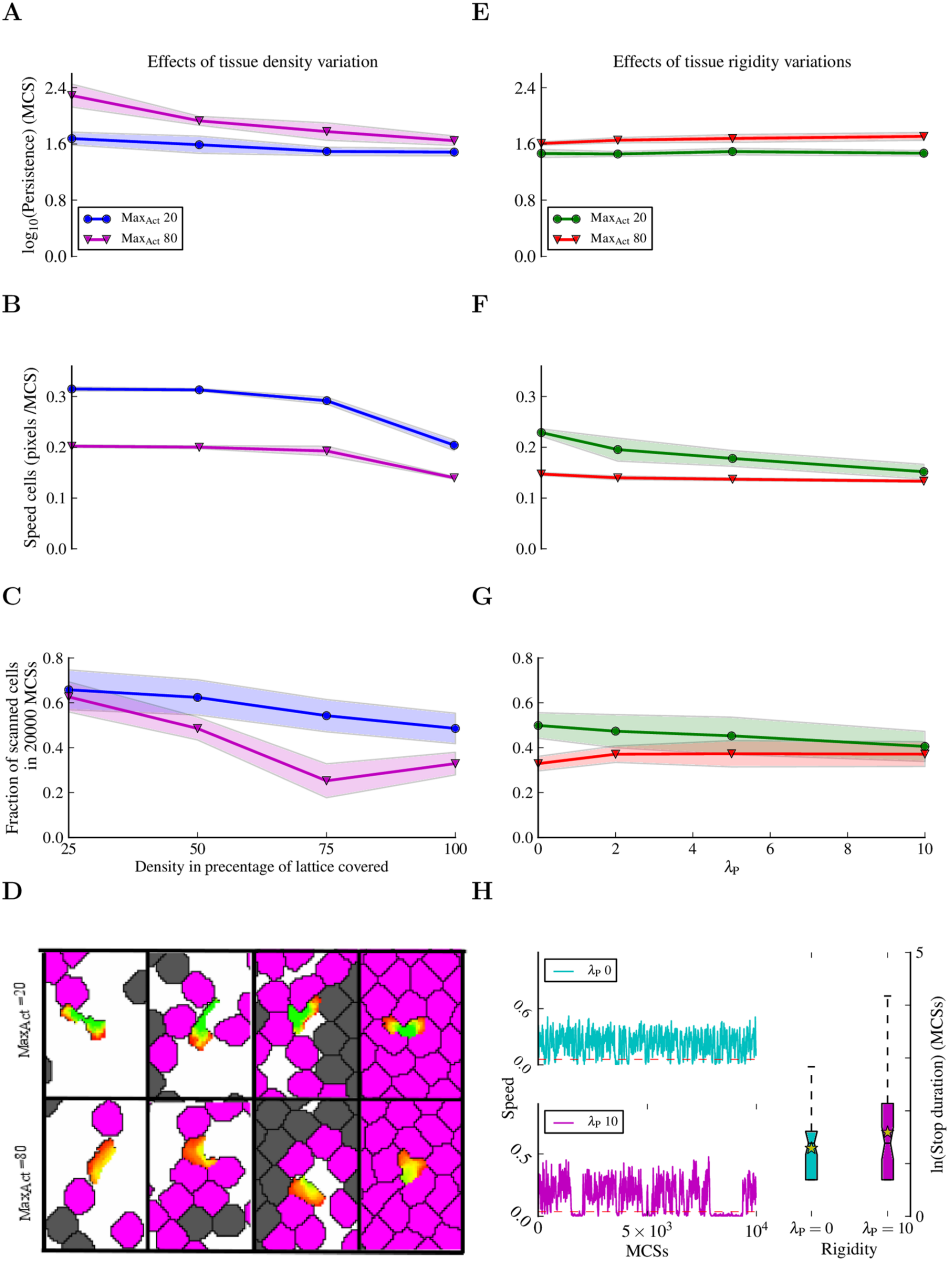
Influence of environment on the migration and scanning properties of Act cells. Effects of variations in tissue density (A-D) and in tissue rigidity (E-H) on migrating cells within the amoeboid (Max_Act_ = 20) and keratocyte-like (Max_Act_ = 80) parameter range. The results represent the average of 20 experiments of 20000 MCSs each, with a sampling time Δ*t* = 20 MCSs between consecutive measurements of cell position. In each experiment, one Act cell was placed on a dish containing non-Act cells seeded at different densities (A-D), or on a dish with non-Act cells at 100% density with different rigidities (E-H). The rigidity of the tissue formed by non-Act cells is controlled by the perimeter constraint (*λ*_Perimeter_). The higher *λ*_Perimeter_, the stricter cells keep to their target perimeter, and the less they tend to deform. (A, E) Migration persistence. (B, F) Instantaneous speed. (C, G) The fraction of tissue cells scanned by the Act cell. A cell is scanned if it has been in contact with the Act cell at least once. (D) Typical morphologies of Act cells (green with yellow to red protrusions) at different tissue densities. The tissue cells that have been scanned by the Act cell are colored magenta, non-scanned cells are gray. (H) (Left) Speed traces of two cells in the amoeboid range (Max_Act_ = 20), one migrating in flexible tissue (*λ*_Perimeter_ = 0) and the other one in rigid tissue (*λ*_Perimeter_ = 10). The red dashed lines delimit the speed thresholds under which the cells were considered stationary. (Right) Boxplots of the stop durations (in MCSs) corresponding to the speed traces in the left panel. Yellow stars indicate means. See Methods section for a more detailed explanation of the experiments and for the complete list of parameter values.

To study the effect of the environment, we explored the migration and scanning properties of amoeboid and keratocyte-like cells in tissues with different densities and rigidities (see Methods and Fig 11). Increasing the environment density decreases the persistence of keratocyte-like cells (Fig 11A), and a very dense tissue (100% lattice coverage) decreases the speed of both types of cells (Fig 11B). Amoeboid cells are better at scanning the environment than keratocyte-like cells, regardless of the tissue density (Fig 11C). We observed an improvement in the scanning capacity of the keratocyte-like cells in very dense environment (Fig 11C). Interestingly, this is caused by a more amoeboid behavior of the cells, which is induced by the environment (Fig 11D and S11 Video).

Increasing tissue rigidity does not affect the persistence of the cells (Fig 11E), but it does cause a slight decrease in amoeboid cell speed (Fig 11F), which is reflected in a rigidity dependent decrease in scanning capacity of these cells (Fig 11G). A very rigid environment promotes a distinct stop and go behavior in amoeboid cells (Fig 11H), which is not detected in more deformable tissue.

In conclusion, the Act model is suited for both simple multicellular systems made up of migrating cells of the same type, as well as for complex multicellular systems populated by cells of different types. Our exploratory analyses provide insight into the conditions needed for collective cell migration and show the critical impact of the environment on the migration of single cells. Furthermore, they provide guidelines for future use as they highlight the properties of the Act model in a broad range of settings.

### Computational complexity

Adding a new mechanism to an existing model comes at a computational cost. To assess both theoretically and empirically the effect of the Act model on the performance of the original CPM, we evaluated the asymptotic computational complexity of the system, and measured CPU time for several simulations with and without the Act model.

One strength of the CPM is its capability to handle all cell properties locally, at the level of single copy attempts from one lattice site to another. In a CPM simulation using a 2D square lattice of width ℓ, ℓ^2^ copy attempts of lattice size independent computation time are performed during each MCS, hence, the asymptotic computational complexity of one MCS is $O(\ell^{2})$. Because the Act model is also entirely based on local information, adding it to the CPM preserves the asymptotic complexity of the system, increasing only the time required to evaluate one copy attempt.

**Fig 12:**
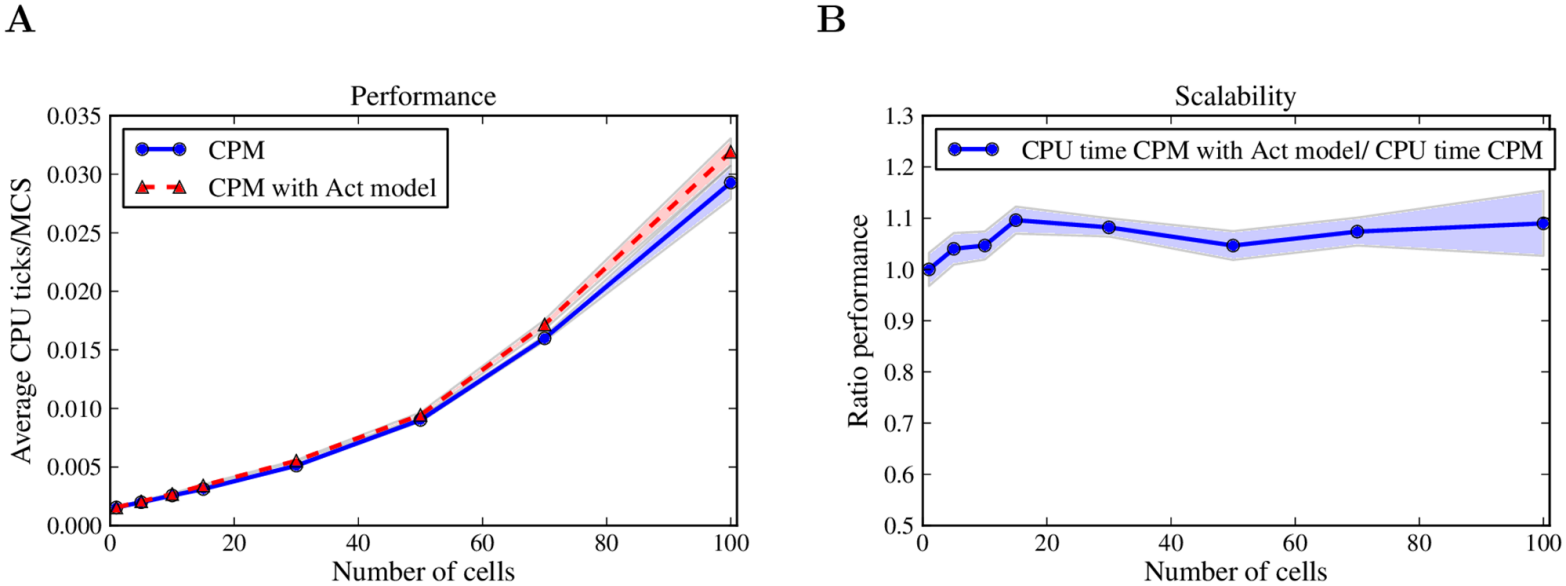
Computational performance of the Act model. (A) The CPU time of the CPM with and without the Act model was measured for simulations of migrating keratocytes (Fig 9) during 3000 MCSs. The computational performance is calculated as CPU time per MCS, averaged over 5 simulations. (B) The ratio performance is the ratio of the performance of the CPM with the Act model to the performance of the CPM alone (at the same number of cells). The performance of the Act model is comparable with that of the basic CPM. The Act model increases the CPU time of the CPM by around 10% and this percentage of extra cost remains constant with increasing cell numbers. Shadows represent the standard deviation. See Methods section for the complete list of parameter values.

In practice, the computation time of one MCS changes not only with the size of the lattice, but also with number of cells on the lattice. CPM implementations are optimized by discarding or completely avoiding trivial copy attempts within the same cell or within the medium, practically acting only at cell borders. Adding cells on the lattice increases the number of cell borders where the non-trivial and computationally more expensive copy attempts take place. Therefore, increasing the number of cells leads to an increase in the computational cost of one MCS. We empirically evaluated the cost of the Act model in such a setting by measuring the CPU time of the CPM with and without the Act model for an increasing number of cells on a fixed size lattice (Fig 12). In these simulations, the Act model increased the CPU time of the system by about 10%, and this overhead did not change appreciably with the number of cells.

In conclusion, enriching the CPM with the Act model preserves its asymptotic computational complexity and increases its computational cost merely by a small percentage. Importantly, this percentage remains constant for large multicellular simulations.

### Conclusion

Within the widely used CPM framework, we have created the Act model based on an actin-inspired mechanism. In the new model, cell shape and motility emerge from the interplay between stochasticity, local positive feedback, and membrane tension. Act model cells break symmetry randomly and move in an amoeboid or keratocyte-like manner. When placed in a chemokine gradient, the *in silico* amoeboid cells reproduce several qualitative features of real chemotaxing amoeboids: they form more protrusions up the chemokine gradient and they react proportionally to the steepness of the gradient. In a multicellular context, *in silico* keratocyte-like cells reproduce the density dependent collective migration of real keratocytes [35], while amoeboid cells are able to squeeze themselves through densely packed tissues, resembling effector T cells crawling through the skin epidermis [7]. The Act model is computationally light, with its computational complexity scaling well with the number of cells on the lattice.

Our model is conceptually related to existing single-cell models that reproduce amoeboid and keratocyte-like cell types [8, 14]. Within a phenomenological framework similar to the CPM, Nishimura et al.[14] obtained cell migration by using the interaction between an actin surrogate and a generic actin inhibitor. In their model, cell deformations create regions of low concentrations of inhibitor which trigger actin surrogate accumulation and further deformations. This results in a feedback mechanism that drives cell polarization and movement. The Act model reproduces the same rich qualitative behavior with a much simpler implementation of actin-like dynamics—it uses two extra CPM parameters versus the six migration related parameters in the Nishimura model—and is sufficiently light to be used in various multicellular contexts.

Multicellular models typically simplify cell shape and cell migration mechanisms in order to reduce computational complexity. For instance, in a 3D CPM model of T cell migration in lymph nodes, Beltman et al. [36] implemented cell movement by steering cells along direction vectors that are periodically updated to the recent cell displacement. This mechanism also results in persistent random migration, and has a similar computational complexity to that of the Act model, however, it cannot reproduce amoeboid behavior because the cells remain roundish. Importantly, in their model cell migration is imposed, while in the Act model cell migration emerges from the changes in cell shape.

A limitation of the Act model is its phenomenological character, which allows only a simplified representation of the actin network and its dynamics. The Act model is implemented within the CPM, which is inherently phenomenological, although there are ongoing efforts to relate the parameters of CPM to biophysical properties of cells [32]. The forces acting upon CPM cells are implicit, controlled globally by the Hamiltonian, and cannot be imposed explicitly at the level of the cell. This means that most cell features can only be inferred by measurements performed during/after simulations. Therefore, the calibration of *in silico* cells amounts to finding the set of parameter values that results in the desired shape, biophysical, and/or migratory cell properties, with prior knowledge on how each CPM parameter influences certain cell properties (see also [37], part II). Noteworthy, the migration features of *in silico* cells in a non-empty environment are partly determined by the environment and therefore they typically cannot be set a priori, but need to be evaluated from simulations. This holds true for all phenomenological migration models proposed thus far.

In the present implementation, the Act model resides on a 2D grid, making it especially suitable for simulating migration on flat surfaces or in very thin tissues, like epithelia. Adapting the model to 3D is straightforward and would facilitate the study of different 3D phenomena like amoeboid migration in soil, the individual or collective migration of cancer cells during metastasis, and lymphocyte migration in organs and tumors.

In conclusion, our simple model is actin-inspired, achieves emergent amoeboid and keratocyte-like behavior with only two additional parameters and reproduces directed and persistent random migration. Because the Act model is light and simple, it can readily be used in large and heterogeneous multicellular simulations, while preserving the shape and behavior of the single-cell.

## Methods

### Model description

The Cellular Potts Model (CPM) [38] is a powerful lattice-based method for modeling cells and tissue dynamics, while retaining individual cell identity. CPM cells occupy areas (i.e, they are collections of lattice sites) on a 2D lattice, and interact with each other and the medium in a way that resembles cell-cell and cell-medium adhesion. Cells “move” by randomly copying their identity into neighboring lattice sites in order to minimize a global energy function, i.e., the Hamiltonian H, that includes the cell-cell and cell-medium interactions and other cell constraints like cell area and cell perimeter:

$$H=\sum_{u,v}J_{\tau \left(\sigma_{u}\right),\tau \left(\sigma_{v}\right)}\left(1-\delta_{\sigma_{u},\sigma_{v}}\right)+\sum_{\sigma }\lambda_{\text{Area}}{\left(a_{\sigma }-A_{\sigma }\right)}^{2}+\sum_{\sigma }\lambda_{\text{Perimeter}}{\left(p_{\sigma }-P_{\sigma }\right)}^{2}$$

where *u* and *v* are neighboring lattice sites, *σ* is the cell identity and *τ* is the cell type. The first term of the Hamiltonian sums the energy values, *J*_*τ*(*σ*_*u*_),*τ*(*σ*_*v*_)_, between all the neighboring lattice sites *u* and *v*, where the term 1 − *δ*_*σ*_*u*_,*σ*_*v*__ (with Kronecker delta *δ*_*σ*_*u*_,*σ*_*v*__ = 1 when *σ*_*u*_ = *σ*_*v*_; and 0 otherwise) ensures that only interactions between different cells are considered in the Hamiltonian. The second term (the area constraint) and the third term (the perimeter constraint) sum over all cells; the system is penalized when the current cell area (*a*_*σ*_) deviates from the target cell area (*A*_*σ*_) or when the current cell perimeter (*p*_*σ*_) deviates from the target cell perimeter (*P*_*σ*_). The parameters *λ*_Area_ and *λ*_Perimeter_ are generally interpreted as resistance to compression and stiffness of the membrane, respectively. The area of a cell is the number of lattice sites with the same identity *σ*, while the perimeter is calculated as the number of distinct interfaces (edges and corners of lattice sites) with neighboring lattice sites of different cells or of the medium [39].

The lattice is updated asynchronously and one Monte Carlo step (MCS) is defined as the number of random copy attempts equal to the number of lattice sites. During each copy attempt, the algorithm picks a random site *u* and one of its neighbors *v* and, if they do not belong to the same cell (i.e., *σ*_*u*_ ≠ *σ*_*v*_), it tries to copy the cell identity of the site *u* into site *v*, effectively trying to allocate site *v* to the cell *σ*_*u*_. In practice, the global value of H need not be calculated for every copy attempt; instead, the energy difference, ΔH, between the current configuration and the potential novel configuration is calculated from local information around *u* and *v*. A copy attempt is always accepted if it decreases the energy of the system (ΔH<0), otherwise it is accepted with the Boltzmann probability $e^{-\Delta H/\text{T}}$, where T is the “temperature” of the system.

Within the CPM, chemotaxis is implemented by increasing the probability of copying to sites with relatively high chemoattractant concentrations: $\Delta H_{\text{Chemotaxis}}\left(u\to v\right)=\lambda_{\text{Chemotaxis}}\left(C_{v}-C_{u}\right)$, where *C*_*u*_ and *C*_*v*_ are the chemoattractant concentrations at lattice sites *u* and *v*. In order to contribute to the energy value of the system, $\Delta H_{\text{Chemotaxis}}$ is subtracted from ΔH. In general, CPM can incorporate various other processes by subtracting additional terms from the ΔH. For an in-depth overview of CPM and its applications see [37].

We have implemented our model in the Tissue Simulation Toolkit, which is a two dimensional open source library for the CPM [19]. Additionally, an on-line interactive implementation is available at http://bioinformatics.bio.uu.nl/ioana/cpm/ (tested in Firefox and Chrome browsers).

### Simulation set-up and parameters

All simulations are two dimensional and use a rectangular lattice. The CPM parameters that have constant values across simulations are the temperature *T* = 20, the area constraint *λ*_Area_ = 50 and the energy value of the CPM medium with itself, *J*_medium,medium_ = 0. The perimeter constraint of all cells is *λ*_Perimeter_ = 2, with the exception of the tissue rigidity experiments where this value is varied for the tissue cells. In the multicellular simulations, we have used the connectivity constraint described by Merks et al. [19] to keep the Act cells from breaking.

The migrating cells in the single cell simulations (Figs 2, 3 and 4) and collective migration simulations (Fig 9) have a constant target area of *A*_cell_ = 500, with *λ*_Area_ = 50 and a target perimeter of *P* = 340. The energy between cells is *J*_cell,cell_ = 100 and the energy between the cells and the medium is *J*_cell,medium_ = 20. The simulations without chemotaxis use a wrapped lattice of size 200×200; the simulations with chemotaxis use a wrapped lattice of size 100×300 and a linear chemoattractant concentration with a slope of 0.33 applied in the y direction (i.e., concentrations *C*_*x*,*y* = 0_ = 0 and *C*_*x*,*y* = 300_ = 100).

In the collective migration experiments (Fig 9), we place Act cells of the same type on a wrapped lattice of size 500x500 and performed simulations in which we varied the cell type, the fraction of lattice coverage, and the cell-cell adhesion. To vary the cell type, we varied the parameter Max_Act_, such that it covers the amoeboid to keratocyte-like range. We varied the fraction of lattice coverage between zero and one, where zero means that the lattice is empty and one that the lattice is full. To vary cell-cell adhesion, we varied the surface tension *γ*_cell,medium_ = *J*_cell,medium_ − (*J*_cell,cell_ + *J*_medium,medium_)/2; a positive *γ*_cell,medium_ encourages cells to “stick” together, while at *γ*_cell,medium_ = 0 cells adhere as much to the medium as to each other, i.e, a neutral value of adhesion. We varied *γ*_cell,medium_ by changing only the energy between the Act cells, *J*_cell,cell_, while keeping *J*_cell,medium_ = 20 and *J*_medium,medium_ = 0 constant. *J*_cell,cell_ = 40 results in *γ*_cell,medium_ = 0, the neutral adhesion value, *J*_cell,cell_ = −60 results in *γ*_cell,medium_ = 50, which we define as a medium adhesion, and *J*_cell,cell_ = −160 results in *γ*_cell,medium_ = 100, which we refer to as high adhesion. The target area of the Act cells is Area_cell_ = 200 and their target perimeter *λ*_Perimeter_ = 180.

In the skin model simulation (Fig 10), we created T cells by using the Act model parameters for amoeboid cells (Max_Act_ = 20, *λ*_Act_ = 2000). The target area of T cells is *A*_Tcell_ = 100 and the target perimeter is *P*_Tcell_ = 140. We created almost inflexible and almost stationary skin cells (the gray cells) by giving basic CPM cells (without the Act mechanism) a tight target perimeter (*P*_skin_ = 145) compared to their target area (*A*_skin_ = 152), which prevents them from ruffling. The energy values in the simulation are *J*_skin,skin_ = *J*_skin,Tcell_ = *J*_skin,medium_ = *J*_skin,medium_ = 20, *J*_Tcell,Tcell_ = 100.

For the exploratory tissue experiments (Fig 11), we performed simulations by placing one Act cell (Max_Act_ = 20 or Max_Act_ = 80 with *λ*_Act_ = 2000) in tissues with different densities, or in tissues with different rigidities at the highest density (a fraction of lattice coverage of 1). The target area and the target perimeter of tissue cells are the same as those of the skin cells in Fig 10, and the target area and target perimeter of the Act cells are the same as those of the T cells in Fig 10. The energy values in the simulations are *J*_tissue,tissue_ = *J*_tissue,ActCell_ = *J*_tissue,medium_ = *J*_ActCell,medium_ = 20, *J*_ActCell,ActCell_ = 100. We have varied the density of the tissue (Fig 11 left column) from relatively scattered cells (a fraction of lattice coverage of 0.25) to a full lattice (a fraction of lattice coverage of 1). We have varied the rigidity of the tissue (Fig 11 right column) by varying the value of *λ*_Perimeter_ of the tissue cells. This parameter governs how stringent a cell conserves its target perimeter: at *λ*_Perimeter_ = 0 a cell ignores the perimeter constraint, while at a higher value of *λ*_Perimeter_ the cell is allowed less deviations from the target perimeter. A higher value of *λ*_Perimeter_ combined with a relative short target perimeter creates cells that are more difficult to deform, hence they are more rigid.

### Measurements

Centroids and principal axes of cells are calculated from the moments of inertia [40]. The length of a cell is the length of its major axis. The instantaneous speed of a cell is the Euclidean distance traveled by its centroid during one MCS. The orientation-direction angle (∈ [0.90]) is the angle between the cell’s longest axis and its direction of migration. The turning angle is the angle between two consecutively measured directions of migration. Persistence time is expressed as the number of MCSs required for a cell to lose the angular correlation of the direction of migration [41]. The motility coefficient is calculated by fitting Fürth’s equation for persistent random migration [42, 43] to mean square displacement plots: $\bar{x^{2}}=2nM\left(t-P\left(1-e^{-t/P}\right)\right)$, where $\bar{x^{2}}$ is the mean square displacement, *n* is the spatial dimension (here *n* = 2), *M* is the motility coefficient, *P* is the persistence time and *t* is the elapsed time since the start of the trajectory.

For the analysis of the cell chemotaxis simulations, we use the directed and scaled directed speed as measurements for chemotaxis sensitivity. The directed speed is defined as the magnitude of the cell velocity projected onto the chemokine gradient. The chemotactic index is defined as the distance traveled up the chemokine gradient divided by the total distance traveled.

A cell protrusion is defined as a connected patch of at least 10 active lattice sites. The orientation of a protrusion relative to the chemokine gradient is defined as the angle between the chemokine gradient and the vector that connects the centroid of the cell with the centroid of the protrusion.

The collectivity of cell migration is calculated as the order index, $\left|\sum_{i=1}^{n}\frac{\vec{v_{i}}}{|v_{i}|}\right|$, where *n* is the number of cells on the lattice and $\vec{v_{i}}$ is the direction vector of cell *i* during one MCS. In order to ensure the evaluation of collectivity at steady state, the order index of one simulation is calculated at the end of 500.000 MCSs.

## Supporting Information

S1 Video Act cell breaks symmetry and polarizes. (MPG)

S2 Video Morphospace video of the amoeboid to keratocyte transition. (MP4)

S3 Video Act cell performing amoeboid migration. (MPG)

S4 Video Act cell performing keratocyte-like migration. (MPG)

S5 Video Amoeboid cell following a vertical chemotactic gradient. (MPG)

S6 Video Keratocyte-like cell following a vertical chemotactic gradient. (MPG)

S7 Video An amoeboid T-cell (green cell with yellow to red protrusions) is patrolling the skin (gray cells). (MPG)

S8 Video Amoeboid cells do not migrate collectively at the highest lattice density. (MP4)

S9 Video Keratocyte-like cells tend to create vortices at high cell-cell adhesion. (MP4)

S10 Video Collective migration of keratocyte-like cells. (MP4)

S11 Video Keratocyte-like cell scanning a dense tissue. A cell in the keratocyte-like range (green with yellow to red protrusions) scans tissue (gray cells). A scanned tissue cell is colored magenta. The dense tissue makes the scanning cell deform and become more amoeboid-like.
(MP4)

S1 Code (ZIP)
