# Supplementary material for: Crawling and Gliding: A Computational Model for Shape-Driven Cell Migration
Source: PLoS Comput Biol. 2015 Oct 21;11(10):e1004280. doi: 10.1371/journal.pcbi.1004280 (PMC4619082; doi:10.1371/journal.pcbi.1004280)
Supplement: S1 Code — (ZIP) [file pcbi.1004280.s012.zip › release/tst/doc/html/qtgraph_8h.html]

Tissue Simulation Toolkit: qtgraph.h File Reference


|  |
| --- |
| Tissue Simulation Toolkit  0.1.4.1 |


- Main Page
- Namespaces
- Classes
- Files

- File List
- File Members

Classes |
Macros

qtgraph.h File Reference

`#include <qwidget.h>`  
`#include <qlabel.h>`  
`#include <qpainter.h>`  
`#include <q3picture.h>`  
`#include <qpixmap.h>`  
`#include <QMouseEvent>`  
`#include <QPaintEvent>`  
`#include <QResizeEvent>`  
`#include "graph.h"`  
`#include <qapplication.h>`

Include dependency graph for qtgraph.h:

This graph shows which files directly or indirectly include this file:

Go to the source code of this file.

|  |  |
| --- | --- |
| Classes | |
| class | QtGraphics |
|  | |

|  |  |
| --- | --- |
| Macros | |
| #define | TIMESTEP   void QtGraphics::TimeStep(void) |
|  | |

## Macro Definition Documentation

|  |
| --- |
| #define TIMESTEP   void QtGraphics::TimeStep(void) |


---

Generated on Thu Aug 14 2014 22:04:01 for Tissue Simulation Toolkit by  

 1.8.6
