# Supplementary material for: Crawling and Gliding: A Computational Model for Shape-Driven Cell Migration
Source: PLoS Comput Biol. 2015 Oct 21;11(10):e1004280. doi: 10.1371/journal.pcbi.1004280 (PMC4619082; doi:10.1371/journal.pcbi.1004280)
Supplement: S1 Code — (ZIP) [file pcbi.1004280.s012.zip › release/tst/doc/html/classParameter.html]

Tissue Simulation Toolkit: Parameter Class Reference


|  |
| --- |
| Tissue Simulation Toolkit  0.1.4.1 |


- Main Page
- Namespaces
- Classes
- Files

- Class List
- Class Hierarchy
- Class Members

Public Member Functions |
Public Attributes |
List of all members

Parameter Class Reference

`#include <parameter.h>`

|  |  |
| --- | --- |
| Public Member Functions | |
|  | Parameter () |
|  | |
|  | ~Parameter () |
|  | |
| void | CleanUp (void) |
|  | |
| void | Read (const char \*filename) |
|  | |
| void | Write (ostream &os) const |
|  | |

|  |  |
| --- | --- |
| Public Attributes | |
| double | T |
|  | |
| int | target\_area |
|  | |
| int | target\_length |
|  | |
| double | lambda |
|  | |
| double | lambda2 |
|  | |
| char \* | Jtable |
|  | |
| int | conn\_diss |
|  | |
| bool | vecadherinknockout |
|  | |
| bool | extensiononly |
|  | |
| int | chemotaxis |
|  | |
| int | border\_energy |
|  | |
| int | neighbours |
|  | |
| bool | periodic\_boundaries |
|  | |
| int | n\_chem |
|  | |
| double \* | diff\_coeff |
|  | |
| double \* | decay\_rate |
|  | |
| double \* | secr\_rate |
|  | |
| double | saturation |
|  | |
| double | dt |
|  | |
| double | dx |
|  | |
| int | pde\_its |
|  | |
| int | n\_init\_cells |
|  | |
| int | size\_init\_cells |
|  | |
| int | sizex |
|  | |
| int | sizey |
|  | |
| int | divisions |
|  | |
| int | mcs |
|  | |
| int | rseed |
|  | |
| double | subfield |
|  | |
| int | relaxation |
|  | |
| int | storage\_stride |
|  | |
| bool | graphics |
|  | |
| bool | store |
|  | |
| char \* | datadir |
|  | |

## Constructor & Destructor Documentation

|  |  |  |  |  |
| --- | --- | --- | --- | --- |
| Parameter::Parameter | ( |  | ) |  |

References border\_energy, chemotaxis, conn\_diss, datadir, decay\_rate, diff\_coeff, divisions, dt, dx, extensiononly, graphics, Jtable, lambda, lambda2, mcs, n\_chem, n\_init\_cells, neighbours, pde\_its, periodic\_boundaries, relaxation, rseed, saturation, secr\_rate, size\_init\_cells, sizex, sizey, storage\_stride, store, subfield, T, target\_area, target\_length, and vecadherinknockout.

|  |  |  |  |  |
| --- | --- | --- | --- | --- |
| Parameter::~Parameter | ( |  | ) |  |

References CleanUp().

## Member Function Documentation

|  |  |  |  |  |  |
| --- | --- | --- | --- | --- | --- |
| void Parameter::CleanUp | ( | void |  | ) |  |

References datadir, decay\_rate, diff\_coeff, Jtable, and secr\_rate.

Referenced by Read(), and ~Parameter().

|  |  |  |  |  |  |
| --- | --- | --- | --- | --- | --- |
| void Parameter::Read | ( | const char \* | *filename* | ) |  |

References bgetpar(), border\_energy, chemotaxis, CleanUp(), conn\_diss, datadir, decay\_rate, dgetparlist(), diff\_coeff, divisions, dt, dx, extensiononly, fgetpar(), graphics, igetpar(), Jtable, lambda, lambda2, mcs, n\_chem, n\_init\_cells, neighbours, OpenReadFile(), pde\_its, periodic\_boundaries, relaxation, rseed, saturation, secr\_rate, sgetpar(), size\_init\_cells, sizex, sizey, storage\_stride, store, subfield, T, target\_area, target\_length, and vecadherinknockout.

|  |  |  |  |  |  |
| --- | --- | --- | --- | --- | --- |
| void Parameter::Write | ( | ostream & | *os* | ) | const |

References border\_energy, chemotaxis, conn\_diss, datadir, decay\_rate, diff\_coeff, divisions, dt, dx, extensiononly, graphics, Jtable, lambda, lambda2, mcs, n\_chem, n\_init\_cells, neighbours, pde\_its, periodic\_boundaries, relaxation, rseed, saturation, sbool(), secr\_rate, size\_init\_cells, sizex, sizey, storage\_stride, store, subfield, T, target\_area, target\_length, and vecadherinknockout.

Referenced by operator<<().

## Member Data Documentation

|  |
| --- |
| int Parameter::border\_energy |

Referenced by Parameter(), Read(), and Write().

|  |
| --- |
| int Parameter::chemotaxis |

Referenced by Parameter(), Read(), and Write().

|  |
| --- |
| int Parameter::conn\_diss |

Referenced by CellularPotts::AmoebaeMove(), Parameter(), Read(), and Write().

|  |
| --- |
| char\* Parameter::datadir |

Referenced by CleanUp(), Parameter(), Read(), and Write().

|  |
| --- |
| double\* Parameter::decay\_rate |

Referenced by CleanUp(), Parameter(), Read(), and Write().

|  |
| --- |
| double\* Parameter::diff\_coeff |

Referenced by CleanUp(), PDE::Diffuse(), Parameter(), Read(), and Write().

|  |
| --- |
| int Parameter::divisions |

Referenced by Parameter(), Read(), and Write().

|  |
| --- |
| double Parameter::dt |

Referenced by PDE::Diffuse(), Parameter(), Read(), and Write().

|  |
| --- |
| double Parameter::dx |

Referenced by PDE::Diffuse(), Parameter(), Read(), and Write().

|  |
| --- |
| bool Parameter::extensiononly |

Referenced by Parameter(), Read(), and Write().

|  |
| --- |
| bool Parameter::graphics |

Referenced by X11Graphics::EndScene(), X11Graphics::Field(), X11Graphics::GetXYCoo(), Parameter(), X11Graphics::Point(), Read(), Write(), and X11Graphics::X11Graphics().

|  |
| --- |
| char\* Parameter::Jtable |

Referenced by CleanUp(), Parameter(), Read(), and Write().

|  |
| --- |
| double Parameter::lambda |

Referenced by Info::Menu(), Parameter(), Read(), and Write().

|  |
| --- |
| double Parameter::lambda2 |

Referenced by Parameter(), Read(), and Write().

|  |
| --- |
| int Parameter::mcs |

Referenced by Parameter(), Read(), QtGraphics::TimeStepWrap(), and Write().

|  |
| --- |
| int Parameter::n\_chem |

Referenced by Cell::Cell(), Cell::CellBirth(), Dish::Dish(), PDE::GradC(), Dish::MeasureChemConcentrations(), Cell::operator=(), Parameter(), Read(), and Write().

|  |
| --- |
| int Parameter::n\_init\_cells |

Referenced by Parameter(), Read(), and Write().

|  |
| --- |
| int Parameter::neighbours |

Referenced by CellularPotts::BaseInitialisation(), CellularPotts::CellularPotts(), Parameter(), Read(), and Write().

|  |
| --- |
| int Parameter::pde\_its |

Referenced by Parameter(), Read(), and Write().

|  |
| --- |
| bool Parameter::periodic\_boundaries |

Referenced by CellularPotts::AmoebaeMove(), PDE::Diffuse(), Parameter(), Read(), and Write().

|  |
| --- |
| int Parameter::relaxation |

Referenced by Parameter(), Read(), and Write().

|  |
| --- |
| int Parameter::rseed |

Referenced by Parameter(), Read(), and Write().

|  |
| --- |
| double Parameter::saturation |

Referenced by Parameter(), Read(), sat(), and Write().

|  |
| --- |
| double\* Parameter::secr\_rate |

Referenced by CleanUp(), Parameter(), Read(), and Write().

|  |
| --- |
| int Parameter::size\_init\_cells |

Referenced by Info::Menu(), Parameter(), Read(), and Write().

|  |
| --- |
| int Parameter::sizex |

Referenced by Dish::Dish(), Parameter(), Read(), and Write().

|  |
| --- |
| int Parameter::sizey |

Referenced by Dish::Dish(), Parameter(), Read(), and Write().

|  |
| --- |
| int Parameter::storage\_stride |

Referenced by X11Graphics::EndScene(), Parameter(), Read(), and Write().

|  |
| --- |
| bool Parameter::store |

Referenced by Parameter(), Read(), Write(), and X11Graphics::X11Graphics().

|  |
| --- |
| double Parameter::subfield |

Referenced by Parameter(), Read(), and Write().

|  |
| --- |
| double Parameter::T |

Referenced by CellularPotts::BaseInitialisation(), CellularPotts::CellularPotts(), Parameter(), Read(), and Write().

|  |
| --- |
| int Parameter::target\_area |

Referenced by CellularPotts::ConstructInitCells(), Dish::Dish(), CellularPotts::GrowAndDivideCells(), Parameter(), Read(), and Write().

|  |
| --- |
| int Parameter::target\_length |

Referenced by Parameter(), Read(), CellularPotts::ResetTargetLengths(), and Write().

|  |
| --- |
| bool Parameter::vecadherinknockout |

Referenced by Parameter(), Read(), and Write().

---

The documentation for this class was generated from the following files:

- parameter.h
- parameter.cpp


---

Generated on Thu Aug 14 2014 22:04:01 for Tissue Simulation Toolkit by  

 1.8.6
