# Supplementary material for: Crawling and Gliding: A Computational Model for Shape-Driven Cell Migration
Source: PLoS Comput Biol. 2015 Oct 21;11(10):e1004280. doi: 10.1371/journal.pcbi.1004280 (PMC4619082; doi:10.1371/journal.pcbi.1004280)
Supplement: S1 Code — (ZIP) [file pcbi.1004280.s012.zip › release/tst/doc/html/sqr_8h.html]

Tissue Simulation Toolkit: sqr.h File Reference


|  |
| --- |
| Tissue Simulation Toolkit  0.1.4.1 |


- Main Page
- Namespaces
- Classes
- Files

- File List
- File Members

Macros

sqr.h File Reference

This graph shows which files directly or indirectly include this file:

Go to the source code of this file.

|  |  |
| --- | --- |
| Macros | |
| #define | SQR(a)   ((sqrarg=(a)) == 0.0 ? 0.0 : sqrarg\*sqrarg) |
|  | |
| #define | DSQR(a)   ((dsqrarg=(a)) == 0.0 ? 0.0 : dsqrarg\*dsqrarg) |
|  | |

## Macro Definition Documentation

|  |  |  |  |  |  |
| --- | --- | --- | --- | --- | --- |
| #define DSQR | ( |  | a | ) | ((dsqrarg=(a)) == 0.0 ? 0.0 : dsqrarg\*dsqrarg) |

|  |  |  |  |  |  |
| --- | --- | --- | --- | --- | --- |
| #define SQR | ( |  | a | ) | ((sqrarg=(a)) == 0.0 ? 0.0 : sqrarg\*sqrarg) |


---

Generated on Thu Aug 14 2014 22:04:01 for Tissue Simulation Toolkit by  

 1.8.6
