# Supplementary material for: Crawling and Gliding: A Computational Model for Shape-Driven Cell Migration
Source: PLoS Comput Biol. 2015 Oct 21;11(10):e1004280. doi: 10.1371/journal.pcbi.1004280 (PMC4619082; doi:10.1371/journal.pcbi.1004280)
Supplement: S1 Code — (ZIP) [file pcbi.1004280.s012.zip › release/tst/doc/html/classPoint.html]

Tissue Simulation Toolkit: Point Class Reference


|  |
| --- |
| Tissue Simulation Toolkit  0.1.4.1 |


- Main Page
- Namespaces
- Classes
- Files

- Class List
- Class Hierarchy
- Class Members

Public Member Functions |
Public Attributes |
List of all members

Point Class Reference

`#include <hull.h>`

|  |  |
| --- | --- |
| Public Member Functions | |
|  | Point (float xx, float yy) |
|  | |
|  | Point (void) |
|  | |

|  |  |
| --- | --- |
| Public Attributes | |
| float | x |
|  | |
| float | y |
|  | |

## Constructor & Destructor Documentation

|  |  |  |  |  |  |  |  |  |  |  |  |  |  |
| --- | --- | --- | --- | --- | --- | --- | --- | --- | --- | --- | --- | --- | --- |
| |  |  |  |  | | --- | --- | --- | --- | | Point::Point | ( | float | *xx*, | |  |  | float | *yy* | |  | ) |  |  | | inline |

References x, and y.

|  |  |  |  |  |  |  |  |
| --- | --- | --- | --- | --- | --- | --- | --- |
| |  |  |  |  |  |  | | --- | --- | --- | --- | --- | --- | | Point::Point | ( | void |  | ) |  | | inline |

References x, and y.

## Member Data Documentation

|  |
| --- |
| float Point::x |

Referenced by chainHull\_2D(), CellularPotts::Compactness(), CellularPotts::DrawConvexHull(), isLeft(), and Point().

|  |
| --- |
| float Point::y |

Referenced by CellularPotts::Compactness(), CellularPotts::DrawConvexHull(), isLeft(), and Point().

---

The documentation for this class was generated from the following file:

- hull.h


---

Generated on Thu Aug 14 2014 22:04:01 for Tissue Simulation Toolkit by  

 1.8.6
