# Supplementary material for: Crawling and Gliding: A Computational Model for Shape-Driven Cell Migration
Source: PLoS Comput Biol. 2015 Oct 21;11(10):e1004280. doi: 10.1371/journal.pcbi.1004280 (PMC4619082; doi:10.1371/journal.pcbi.1004280)
Supplement: S1 Code — (ZIP) [file pcbi.1004280.s012.zip › release/tst/doc/html/namespacemembers.html]

Tissue Simulation Toolkit: Namespace Members


|  |
| --- |
| Tissue Simulation Toolkit  0.1.4.1 |


- Main Page
- Namespaces
- Classes
- Files

- Namespace List
- Namespace Members

- All
- Enumerator

Here is a list of all namespace members with links to the namespace documentation for each member:

- Auxilliary
  : ColourMode
- CellType
  : ColourMode
- Sigma
  : ColourMode
- State
  : ColourMode


---

Generated on Thu Aug 14 2014 22:04:01 for Tissue Simulation Toolkit by  

 1.8.6
