# Supplementary material for: Crawling and Gliding: A Computational Model for Shape-Driven Cell Migration
Source: PLoS Comput Biol. 2015 Oct 21;11(10):e1004280. doi: 10.1371/journal.pcbi.1004280 (PMC4619082; doi:10.1371/journal.pcbi.1004280)
Supplement: S1 Code — (ZIP) [file pcbi.1004280.s012.zip › release/tst/doc/html/classCell-members.html]

Tissue Simulation Toolkit: Member List


|  |
| --- |
| Tissue Simulation Toolkit  0.1.4.1 |


- Main Page
- Namespaces
- Classes
- Files

- Class List
- Class Hierarchy
- Class Members

Cell Member List

This is the complete list of members for Cell, including all inherited members.

|  |  |  |
| --- | --- | --- |
| AddToGrad(double \*g) | Cell | inline |
| alive | Cell | protected |
| AliveP(void) const | Cell | inline |
| amount | Cell | protectedstatic |
| Apoptose() | Cell | inline |
| area | Cell | protected |
| Area() const | Cell | inline |
| capacity | Cell | protectedstatic |
| Cell(const Dish &who, int settau=1) | Cell | inline |
| Cell(void) | Cell | inline |
| Cell(const Cell &src) | Cell | inline |
| CellBirth(Cell &mother) | Cell |  |
| CellularPotts class | Cell | friend |
| chem | Cell | protected |
| ClearGrad(void) | Cell | inline |
| ClearJ(void) | Cell | static |
| Colour(void) const | Cell | inline |
| colour | Cell | protected |
| colour\_of\_birth | Cell | protected |
| ColourOfBirth(void) const | Cell | inline |
| date\_of\_birth | Cell | protected |
| DateOfBirth(void) const | Cell | inline |
| daughter | Cell | protected |
| Daughter(void) const | Cell | inline |
| DecrementTargetArea() | Cell | inline |
| Dish class | Cell | friend |
| EnergyDifference(const Cell &cell2) const | Cell |  |
| GetGrad(void) const | Cell | inline |
| GetJ(const Cell &c2) const | Cell | inline |
| getTau(void) | Cell | inline |
| grad | Cell | protected |
| GradX() const | Cell | inline |
| GradY() const | Cell | inline |
| growth\_threshold | Cell | protected |
| IncrementTargetArea() | Cell | inline |
| Info class | Cell | friend |
| J | Cell | protectedstatic |
| length | Cell | protected |
| Length(void) | Cell | inline |
| MaxSigma() | Cell | inlinestatic |
| maxsigma | Cell | protectedstatic |
| maxtau | Cell | protectedstatic |
| MeasureCellSize(Cell &c) | Cell |  |
| mother | Cell | protected |
| Mother(void) const | Cell | inline |
| n\_copies | Cell | protected |
| operator=(const Cell &src) | Cell | inline |
| owner | Cell | protected |
| polarvec | Cell |  |
| PrintInertia(void) | Cell | inline |
| RenormPolarVec(void) | Cell |  |
| SetColour(const int new\_colour) | Cell | inline |
| SetGrad(double \*g) | Cell | inline |
| SetJ(int t1, int t2, int val) | Cell | inlinestatic |
| SetTargetArea(const int new\_area) | Cell | inline |
| SetTargetLength(double l) | Cell | inline |
| setTau(int settau) | Cell | inline |
| Sigma() const | Cell | inline |
| sigma | Cell | protected |
| sum\_x | Cell | protected |
| sum\_xx | Cell | protected |
| sum\_xy | Cell | protected |
| sum\_y | Cell | protected |
| sum\_yy | Cell | protected |
| target\_area | Cell | protected |
| target\_length | Cell | protected |
| TargetArea() const | Cell | inline |
| TargetLength() const | Cell | inline |
| tau | Cell | protected |
| times\_divided | Cell | protected |
| TimesDivided(void) const | Cell | inline |
| v | Cell | protected |
| ~Cell(void) | Cell |  |


---

Generated on Thu Aug 14 2014 22:04:01 for Tissue Simulation Toolkit by  

 1.8.6
