# Supplementary material for: Crawling and Gliding: A Computational Model for Shape-Driven Cell Migration
Source: PLoS Comput Biol. 2015 Oct 21;11(10):e1004280. doi: 10.1371/journal.pcbi.1004280 (PMC4619082; doi:10.1371/journal.pcbi.1004280)
Supplement: S1 Code — (ZIP) [file pcbi.1004280.s012.zip › release/tst/doc/html/classDish.html]

Tissue Simulation Toolkit: Dish Class Reference


|  |
| --- |
| Tissue Simulation Toolkit  0.1.4.1 |


- Main Page
- Namespaces
- Classes
- Files

- Class List
- Class Hierarchy
- Class Members

Public Member Functions |
Public Attributes |
Protected Member Functions |
Protected Attributes |
Friends |
List of all members

Dish Class Reference

The virtual Petri dish.
More...

`#include <dish.h>`

Collaboration diagram for Dish:

[legend]

|  |  |
| --- | --- |
| Public Member Functions | |
|  | Dish (void) |
|  | |
| void | Init (void) |
|  | Init defines the initial state of the virtual cell culture. More... |
|  | |
| void | ConstructorBody (void) |
|  | |
| virtual | ~Dish () |
|  | |
| void | Plot (Graphics \*g) |
|  | Plot the Dish to graphics window g. More... |
|  | |
| int | ZygoteArea (void) const |
|  | |
| int | Time (void) const |
|  | Returns the number of completed Monte Carlo Steps. More... |
|  | |
| int | CountCells (void) const |
|  | Returns the number of cells in the dish, excluding apoptosed cells. More... |
|  | |
| void | CellGrowthAndDivision (void) |
|  | Stretched induced cell growth and division. More... |
|  | |
| int | Area (void) const |
|  | . Returns the summed area of all cells in the dish More... |
|  | |
| int | TargetArea (void) const |
|  | Returns the summed of all cells target area in the dish. More... |
|  | |
| int | SizeX (void) |
|  | Returns the horizontal size of the dish. More... |
|  | |
| int | SizeY (void) |
|  | Returns the horizontal size of the dish. More... |
|  | |
| Cell & | getCell (int c) |
|  | Returns a reference to cell number "c". More... |
|  | |
| void | ClearGrads (void) |
|  | |
| void | MeasureChemConcentrations (void) |
|  | |

|  |  |
| --- | --- |
| Public Attributes | |
| PDE \* | PDEfield |
|  | |
| CellularPotts \* | CPM |
|  | |

|  |  |
| --- | --- |
| Protected Member Functions | |
| void | SetCellOwner (Cell &which\_cell) |
|  | Assign a the cell to the current Dish. More... |
|  | |

|  |  |
| --- | --- |
| Protected Attributes | |
| std::vector< Cell > | cell |
|  | The cells in the Petri dish; accessible to derived classes. More... |
|  | |

|  |  |
| --- | --- |
| Friends | |
| class | Info |
|  | |

## Detailed Description

The virtual Petri dish.

Hosts the cells with states and the CA-plane.

## Constructor & Destructor Documentation

|  |  |  |  |  |  |
| --- | --- | --- | --- | --- | --- |
| Dish::Dish | ( | void |  | ) |  |

References Parameter::n\_chem, Parameter::sizex, Parameter::sizey, and Parameter::target\_area.

|  |  |  |  |  |  |  |
| --- | --- | --- | --- | --- | --- | --- |
| |  |  |  |  |  | | --- | --- | --- | --- | --- | | Dish::~Dish | ( |  | ) |  | | virtual |

## Member Function Documentation

|  |  |  |  |  |  |
| --- | --- | --- | --- | --- | --- |
| int Dish::Area | ( | void |  | ) | const |

. Returns the summed area of all cells in the dish

|  |  |  |  |  |  |
| --- | --- | --- | --- | --- | --- |
| void Dish::CellGrowthAndDivision | ( | void |  | ) |  |

Stretched induced cell growth and division.

See Hogeweg (2000), Journal of Theoretical Biology.

Find stretched cells, and increase their target area. Find enlarged cells, and divide them.

References Cell::IncrementTargetArea().

|  |  |  |  |  |  |
| --- | --- | --- | --- | --- | --- |
| void Dish::ClearGrads | ( | void |  | ) |  |

|  |  |  |  |  |  |
| --- | --- | --- | --- | --- | --- |
| void Dish::ConstructorBody | ( | void |  | ) |  |

References Cell::maxsigma.

|  |  |  |  |  |  |
| --- | --- | --- | --- | --- | --- |
| int Dish::CountCells | ( | void |  | ) | const |

Returns the number of cells in the dish, excluding apoptosed cells.

|  |  |  |  |  |  |  |  |
| --- | --- | --- | --- | --- | --- | --- | --- |
| |  |  |  |  |  |  | | --- | --- | --- | --- | --- | --- | | Cell& Dish::getCell | ( | int | *c* | ) |  | | inline |

Returns a reference to cell number "c".

References cell.

|  |  |  |  |  |  |
| --- | --- | --- | --- | --- | --- |
| void Dish::Init | ( | void |  | ) |  |

Init defines the initial state of the virtual cell culture.

Define Init() in your main file describing the simulation set up, within the block INIT { }. See for examples vessel.cpp and sorting.cpp.

|  |  |  |  |  |  |
| --- | --- | --- | --- | --- | --- |
| void Dish::MeasureChemConcentrations | ( | void |  | ) |  |

References Cell::Area(), Cell::chem, and Parameter::n\_chem.

|  |  |  |  |  |  |
| --- | --- | --- | --- | --- | --- |
| void Dish::Plot | ( | Graphics \* | *g* | ) |  |

Plot the Dish to graphics window g.

Simply calls CPM->Plot.

|  |  |  |  |  |  |  |  |
| --- | --- | --- | --- | --- | --- | --- | --- |
| |  |  |  |  |  |  | | --- | --- | --- | --- | --- | --- | | void Dish::SetCellOwner | ( | Cell & | *which\_cell* | ) |  | | protected |

Assign a the cell to the current Dish.

References Cell::owner.

|  |  |  |  |  |  |
| --- | --- | --- | --- | --- | --- |
| int Dish::SizeX | ( | void |  | ) |  |

Returns the horizontal size of the dish.

|  |  |  |  |  |  |
| --- | --- | --- | --- | --- | --- |
| int Dish::SizeY | ( | void |  | ) |  |

Returns the horizontal size of the dish.

|  |  |  |  |  |  |
| --- | --- | --- | --- | --- | --- |
| int Dish::TargetArea | ( | void |  | ) | const |

Returns the summed of all cells target area in the dish.

|  |  |  |  |  |  |
| --- | --- | --- | --- | --- | --- |
| int Dish::Time | ( | void |  | ) | const |

Returns the number of completed Monte Carlo Steps.

Referenced by Cell::CellBirth().

|  |  |  |  |  |  |
| --- | --- | --- | --- | --- | --- |
| int Dish::ZygoteArea | ( | void |  | ) | const |

## Friends And Related Function Documentation

|  |  |  |
| --- | --- | --- |
| |  | | --- | | friend class Info | | friend |

## Member Data Documentation

|  |  |  |
| --- | --- | --- |
| |  | | --- | | std::vector<Cell> Dish::cell | | protected |

The cells in the Petri dish; accessible to derived classes.

Referenced by getCell().

|  |
| --- |
| CellularPotts\* Dish::CPM |

|  |
| --- |
| PDE\* Dish::PDEfield |

---

The documentation for this class was generated from the following files:

- dish.h
- dish.cpp


---

Generated on Thu Aug 14 2014 22:04:01 for Tissue Simulation Toolkit by  

 1.8.6
