# Supplementary material for: Crawling and Gliding: A Computational Model for Shape-Driven Cell Migration
Source: PLoS Comput Biol. 2015 Oct 21;11(10):e1004280. doi: 10.1371/journal.pcbi.1004280 (PMC4619082; doi:10.1371/journal.pcbi.1004280)
Supplement: S1 Code — (ZIP) [file pcbi.1004280.s012.zip › release/tst/doc/html/classDish-members.html]

Tissue Simulation Toolkit: Member List


|  |
| --- |
| Tissue Simulation Toolkit  0.1.4.1 |


- Main Page
- Namespaces
- Classes
- Files

- Class List
- Class Hierarchy
- Class Members

Dish Member List

This is the complete list of members for Dish, including all inherited members.

|  |  |  |
| --- | --- | --- |
| Area(void) const | Dish |  |
| cell | Dish | protected |
| CellGrowthAndDivision(void) | Dish |  |
| ClearGrads(void) | Dish |  |
| ConstructorBody(void) | Dish |  |
| CountCells(void) const | Dish |  |
| CPM | Dish |  |
| Dish(void) | Dish |  |
| getCell(int c) | Dish | inline |
| Info class | Dish | friend |
| Init(void) | Dish |  |
| MeasureChemConcentrations(void) | Dish |  |
| PDEfield | Dish |  |
| Plot(Graphics \*g) | Dish |  |
| SetCellOwner(Cell &which\_cell) | Dish | protected |
| SizeX(void) | Dish |  |
| SizeY(void) | Dish |  |
| TargetArea(void) const | Dish |  |
| Time(void) const | Dish |  |
| ZygoteArea(void) const | Dish |  |
| ~Dish() | Dish | virtual |


---

Generated on Thu Aug 14 2014 22:04:01 for Tissue Simulation Toolkit by  

 1.8.6
