# Supplementary material for: Crawling and Gliding: A Computational Model for Shape-Driven Cell Migration
Source: PLoS Comput Biol. 2015 Oct 21;11(10):e1004280. doi: 10.1371/journal.pcbi.1004280 (PMC4619082; doi:10.1371/journal.pcbi.1004280)
Supplement: S1 Code — (ZIP) [file pcbi.1004280.s012.zip › release/tst/doc/html/classGraphics.html]

Tissue Simulation Toolkit: Graphics Class Reference


|  |
| --- |
| Tissue Simulation Toolkit  0.1.4.1 |


- Main Page
- Namespaces
- Classes
- Files

- Class List
- Class Hierarchy
- Class Members

Public Member Functions |
List of all members

Graphics Class Referenceabstract

API for Graphics windows.
More...

`#include <graph.h>`

Inheritance diagram for Graphics:

[legend]

|  |  |
| --- | --- |
| Public Member Functions | |
| virtual | ~Graphics (void) |
|  | |
| virtual void | BeginScene (void) |
|  | BeginScene() must be called before calling drawing functions. More... |
|  | |
| virtual void | EndScene (void) |
|  | EndScene() must be called to flush the drawing buffer and display the scene. More... |
|  | |
| virtual void | Point (int color, int x, int y)=0 |
|  | Plot a point in the Graphics window. More... |
|  | |
| virtual void | Line (int x1, int y1, int x2, int y2, int colour)=0 |
|  | Draws a line (obviously... :-) More... |
|  | |
| virtual int | GetXYCoo (int \*X, int \*Y)=0 |
|  | Probes the Window for user interaction, with mouse or keyboard. More... |
|  | |
| virtual int | XField (void) const |
|  | Returns the width of the Graphics window, in pixels. More... |
|  | |
| virtual int | YField (void) const |
|  | Returns the height of the Graphics window, in pixels. More... |
|  | |
| virtual void | Write (char \*fname, int quality=-1)=0 |
|  | Writes the Image to a file. More... |
|  | |
| virtual void | TimeStep (void) |
|  | Implement this member function in your simulation code. More... |
|  | |
| virtual void | Field (const int \*\*f, int mag=1) |
|  | Plots a field of values given by \*\*f, using color coding given by colormap file. More... |
|  | |

## Detailed Description

API for Graphics windows.

No implementation here. Implemented by X11Graphics and QtGraphics.

## Constructor & Destructor Documentation

|  |  |  |  |  |  |  |  |
| --- | --- | --- | --- | --- | --- | --- | --- |
| |  |  |  |  |  |  | | --- | --- | --- | --- | --- | --- | | virtual Graphics::~Graphics | ( | void |  | ) |  | | inlinevirtual |

## Member Function Documentation

|  |  |  |  |  |  |  |  |
| --- | --- | --- | --- | --- | --- | --- | --- |
| |  |  |  |  |  |  | | --- | --- | --- | --- | --- | --- | | virtual void Graphics::BeginScene | ( | void |  | ) |  | | inlinevirtual |

BeginScene() must be called before calling drawing functions.

Reimplemented in X11Graphics, and QtGraphics.

|  |  |  |  |  |  |  |  |
| --- | --- | --- | --- | --- | --- | --- | --- |
| |  |  |  |  |  |  | | --- | --- | --- | --- | --- | --- | | virtual void Graphics::EndScene | ( | void |  | ) |  | | inlinevirtual |

EndScene() must be called to flush the drawing buffer and display the scene.

Reimplemented in X11Graphics, and QtGraphics.

|  |  |  |  |  |  |  |  |  |  |  |  |  |  |
| --- | --- | --- | --- | --- | --- | --- | --- | --- | --- | --- | --- | --- | --- |
| |  |  |  |  | | --- | --- | --- | --- | | virtual void Graphics::Field | ( | const int \*\* | *f*, | |  |  | int | *mag* = `1` | |  | ) |  |  | | inlinevirtual |

Plots a field of values given by \*\*f, using color coding given by colormap file.

Only implemented in X11Graphics. No checks. Usage not recommended.

Parameters
:   |  |  |
    | --- | --- |
    | f | Double pointer to array of integers, giving color indices using standard colormap ('default.ctb'). |
    | mag | magnification factor. |

Reimplemented in X11Graphics.

|  |  |  |  |  |  |  |  |  |  |  |  |  |  |
| --- | --- | --- | --- | --- | --- | --- | --- | --- | --- | --- | --- | --- | --- |
| |  |  |  |  | | --- | --- | --- | --- | | virtual int Graphics::GetXYCoo | ( | int \* | *X*, | |  |  | int \* | *Y* | |  | ) |  |  | | pure virtual |

Probes the Window for user interaction, with mouse or keyboard.

This function should return immediately, and return 0 if there was no user interaction.

Parameters
:   |  |  |
    | --- | --- |
    | \*X,\*Y | Pointer where the clicked coordinate will be stored. |

Implemented in X11Graphics, and QtGraphics.

Referenced by Info::ClickCell().

|  |  |  |  |  |  |  |  |  |  |  |  |  |  |  |  |  |  |  |  |  |  |  |  |  |  |
| --- | --- | --- | --- | --- | --- | --- | --- | --- | --- | --- | --- | --- | --- | --- | --- | --- | --- | --- | --- | --- | --- | --- | --- | --- | --- |
| |  |  |  |  | | --- | --- | --- | --- | | virtual void Graphics::Line | ( | int | *x1*, | |  |  | int | *y1*, | |  |  | int | *x2*, | |  |  | int | *y2*, | |  |  | int | *colour* | |  | ) |  |  | | pure virtual |

Draws a line (obviously... :-)

Parameters
:   |  |  |
    | --- | --- |
    | x1,y1 | First coordinate pair. |
    | x2,y2 | Second coordinate pair. |
    | color | Color of the line, as given in the colormap file "default.ctb". |

Implemented in X11Graphics, and QtGraphics.

Referenced by CellularPotts::DrawConvexHull(), PDE::PlotVectorField(), and CellularPotts::ShowDirections().

|  |  |  |  |  |  |  |  |  |  |  |  |  |  |  |  |  |  |
| --- | --- | --- | --- | --- | --- | --- | --- | --- | --- | --- | --- | --- | --- | --- | --- | --- | --- |
| |  |  |  |  | | --- | --- | --- | --- | | virtual void Graphics::Point | ( | int | *color*, | |  |  | int | *x*, | |  |  | int | *y* | |  | ) |  |  | | pure virtual |

Plot a point in the Graphics window.

Parameters
:   |  |  |
    | --- | --- |
    | color | Color index, as defined in colormap file "default.ctb", which should be in the same directory as the executable. |
    | x,y | Coordinate of point, in Graphics coordinates (typically twice as large as the cellular automata coordinates). |

Implemented in X11Graphics, and QtGraphics.

Referenced by conrec(), PDE::Plot(), CellularPotts::PlotSigma(), and CellularPotts::SearchNandPlot().

|  |  |  |  |  |  |  |  |
| --- | --- | --- | --- | --- | --- | --- | --- |
| |  |  |  |  |  |  | | --- | --- | --- | --- | --- | --- | | virtual void Graphics::TimeStep | ( | void |  | ) |  | | inlinevirtual |

Implement this member function in your simulation code.

Include all actions that should be carried out during a simulation step, including PDE and CPM simulation steps. See the included examples (vessel.cpp, sorting.cpp) for more information.

Reimplemented in X11Graphics, and QtGraphics.

|  |  |  |  |  |  |  |  |  |  |  |  |  |  |
| --- | --- | --- | --- | --- | --- | --- | --- | --- | --- | --- | --- | --- | --- |
| |  |  |  |  | | --- | --- | --- | --- | | virtual void Graphics::Write | ( | char \* | *fname*, | |  |  | int | *quality* = `-1` | |  | ) |  |  | | pure virtual |

Writes the Image to a file.

File format is inferred from file extension. Currently only PNG is supported by the X-Windows implementation; the Qt-implentation supports all formats supported by Qt.

Parameters
:   |  |  |
    | --- | --- |
    | fname | File name with standard image file extension (e.g. png). |
    | quality | Quality of JPEG images, defaults to -1 (no value provided). |

Implemented in X11Graphics, and QtGraphics.

|  |  |  |  |  |  |  |  |
| --- | --- | --- | --- | --- | --- | --- | --- |
| |  |  |  |  |  |  | | --- | --- | --- | --- | --- | --- | | virtual int Graphics::XField | ( | void |  | ) | const | | inlinevirtual |

Returns the width of the Graphics window, in pixels.

Reimplemented in X11Graphics, and QtGraphics.

|  |  |  |  |  |  |  |  |
| --- | --- | --- | --- | --- | --- | --- | --- |
| |  |  |  |  |  |  | | --- | --- | --- | --- | --- | --- | | virtual int Graphics::YField | ( | void |  | ) | const | | inlinevirtual |

Returns the height of the Graphics window, in pixels.

Reimplemented in X11Graphics, and QtGraphics.

---

The documentation for this class was generated from the following file:

- graph.h


---

Generated on Thu Aug 14 2014 22:04:01 for Tissue Simulation Toolkit by  

 1.8.6
