# Supplementary material for: Crawling and Gliding: A Computational Model for Shape-Driven Cell Migration
Source: PLoS Comput Biol. 2015 Oct 21;11(10):e1004280. doi: 10.1371/journal.pcbi.1004280 (PMC4619082; doi:10.1371/journal.pcbi.1004280)
Supplement: S1 Code — (ZIP) [file pcbi.1004280.s012.zip › release/tst/doc/html/warning_8cpp.html]

Tissue Simulation Toolkit: warning.cpp File Reference


|  |
| --- |
| Tissue Simulation Toolkit  0.1.4.1 |


- Main Page
- Namespaces
- Classes
- Files

- File List
- File Members

Functions |
Variables

warning.cpp File Reference

`#include <stdarg.h>`  
`#include <stdio.h>`  
`#include <stdlib.h>`  
`#include "warning.h"`

Include dependency graph for warning.cpp:

|  |  |
| --- | --- |
| Functions | |
| void | error (char \*fmt,...) |
|  | |
| void | warning (char \*fmt,...) |
|  | |

|  |  |
| --- | --- |
| Variables | |
| int | Quiet =0 |
|  | |

## Function Documentation

|  |  |  |  |
| --- | --- | --- | --- |
| void error | ( | char \* | *fmt*, |
|  |  |  | *...* |
|  | ) |  |  |

Referenced by bgetpar(), CheckFile(), dgetparlist(), ParsePar(), ReadLine(), SearchToken(), and SkipToken().

|  |  |  |  |
| --- | --- | --- | --- |
| void warning | ( | char \* | *fmt*, |
|  |  |  | *...* |
|  | ) |  |  |

References Quiet.

Referenced by bgetpar(), dgetparlist(), fgetpar(), igetpar(), ParsePar(), SearchToken(), and sgetpar().

## Variable Documentation

|  |
| --- |
| int Quiet =0 |

Referenced by warning().


---

Generated on Thu Aug 14 2014 22:04:01 for Tissue Simulation Toolkit by  

 1.8.6
