# Supplementary material for: Crawling and Gliding: A Computational Model for Shape-Driven Cell Migration
Source: PLoS Comput Biol. 2015 Oct 21;11(10):e1004280. doi: 10.1371/journal.pcbi.1004280 (PMC4619082; doi:10.1371/journal.pcbi.1004280)
Supplement: S1 Code — (ZIP) [file pcbi.1004280.s012.zip › release/tst/doc/html/dish_8cpp.html]

Tissue Simulation Toolkit: dish.cpp File Reference


|  |
| --- |
| Tissue Simulation Toolkit  0.1.4.1 |


- Main Page
- Namespaces
- Classes
- Files

- File List
- File Members

Macros |
Variables

dish.cpp File Reference

`#include <vector>`  
`#include <list>`  
`#include <algorithm>`  
`#include <fstream>`  
`#include <string.h>`  
`#include <errno.h>`  
`#include <math.h>`  
`#include "dish.h"`  
`#include "sticky.h"`  
`#include "parameter.h"`  
`#include "info.h"`  
`#include "crash.h"`  
`#include "pde.h"`

Include dependency graph for dish.cpp:

|  |  |
| --- | --- |
| Macros | |
| #define | EXTERNAL\_OFF |
|  | |

|  |  |
| --- | --- |
| Variables | |
| Parameter | par |
|  | |

## Macro Definition Documentation

|  |
| --- |
| #define EXTERNAL\_OFF |

## Variable Documentation

|  |
| --- |
| Parameter par |


---

Generated on Thu Aug 14 2014 22:04:01 for Tissue Simulation Toolkit by  

 1.8.6
