# Supplementary material for: Crawling and Gliding: A Computational Model for Shape-Driven Cell Migration
Source: PLoS Comput Biol. 2015 Oct 21;11(10):e1004280. doi: 10.1371/journal.pcbi.1004280 (PMC4619082; doi:10.1371/journal.pcbi.1004280)
Supplement: S1 Code — (ZIP) [file pcbi.1004280.s012.zip › release/tst/doc/html/pde_8h_source.html]

Tissue Simulation Toolkit: pde.h Source File


|  |
| --- |
| Tissue Simulation Toolkit  0.1.4.1 |


- Main Page
- Namespaces
- Classes
- Files

- File List
- File Members

pde.h

Go to the documentation of this file.

1 /\*

2

3 Copyright 1996-2006 Roeland Merks

4

5 This file is part of Tissue Simulation Toolkit.

6

7 Tissue Simulation Toolkit is free software; you can redistribute

8 it and/or modify it under the terms of the GNU General Public

9 License as published by the Free Software Foundation; either

10 version 2 of the License, or (at your option) any later version.

11

12 Tissue Simulation Toolkit is distributed in the hope that it will

13 be useful, but WITHOUT ANY WARRANTY; without even the implied

14 warranty of MERCHANTABILITY or FITNESS FOR A PARTICULAR PURPOSE.

15 See the GNU General Public License for more details.

16

17 You should have received a copy of the GNU General Public License

18 along with Tissue Simulation Toolkit; if not, write to the Free

19 Software Foundation, Inc., 51 Franklin St, Fifth Floor, Boston, MA

20 02110-1301 USA

21

22 \*/

23

24 #ifndef \_PDE\_HH\_

25 #define \_PDE\_HH\_

26 #include <stdio.h>

27 #include <float.h>

28 #include "graph.h"

29

30 class CellularPotts;

31 class PDE {

32

33  friend class Info;

34

35  public:

36

44  PDE(const int layers, const int sizex,

45  const int sizey);

46

47

48  // destructor must also be virtual

49  virtual ~PDE();

50

56  void Plot(Graphics \*g, const int layer=0);

57

63  void Plot(Graphics \*g, CellularPotts \*cpm, const int layer=0);

64

71  void ContourPlot(Graphics \*g, int layer=0, int colour=1);

72

74  inline int SizeX() const {

75  return sizex;

76  }

77

79  inline int SizeY() const {

80  return sizey;

81  }

82

84  inline int Layers() const {

85  return layers;

86  }

87

95  inline double Sigma(const int layer, const int x, const int y) const {

96  return sigma[layer][x][y];

97  }

98

106  inline void setValue(const int layer, const int x, const int y, const double value) {

107  sigma[layer][x][y]=value;

108  }

109

116  inline void addtoValue(const int layer, const int x, const int y, const double value) {

117  sigma[layer][x][y]+=value;

118  }

119

125  inline double Max(int l) {

126  double max=sigma[l][0][0];

127  int loop=sizex\*sizey;

128  for (int i=1;i<loop;i++)

129  if (sigma[l][0][i]>max) {

130  max=sigma[l][0][i];

131  }

132  return max;

133  }

139  inline double Min(int l) {

140  double min=sigma[l][0][0];

141  int loop=sizex\*sizey;

142  for (int i=1;i<loop;i++)

143  if (sigma[l][0][i]<min) {

144  min=sigma[l][0][i];

145  }

146  return min;

147  }

148

160  void Diffuse(int repeat);

161

165  void NoFluxBoundaries(void);

166

170  void AbsorbingBoundaries(void);

171

175  void PeriodicBoundaries(void);

176

185  void Secrete(CellularPotts \*cpm);

186

189  inline double TheTime(void) const {

190  return thetime;

191  }

192

198  double GetChemAmount(const int layer=-1);

199

210  void GradC(int layer=0, int first\_grad\_layer=1);

211

225  void PlotVectorField(Graphics &g, int stride, int linelength, int first\_grad\_layer=1);

226

227  protected:

228

229  double \*\*\*sigma;

230

231  // Used as temporary memory in the diffusion step

232  // (addresses will be swapped for every time step, so

233  // never directly use them!!! Access is guaranteed to be correct

234  // through user interface)

235

236  double \*\*\*alt\_sigma;

237

238  int sizex;

239  int sizey;

240  int layers;

241

242

243  // Protected member functions

244

251  virtual int MapColour(double val);

252

254  PDE(void);

255

261  virtual double \*\*\*AllocateSigma(const int layers, const int sx, const int sy);

262

263  private:

264  static const int nx[9], ny[9];

265  double thetime;

266

267 };

268

269

270 #endif

PDE::alt\_sigma

double \*\*\* alt\_sigma

**Definition:** pde.h:236

PDE::TheTime

double TheTime(void) const

Returns cumulative "simulated" time, i.e. number of time steps \* dt.

**Definition:** pde.h:189

max

#define max(x, y)

**Definition:** conrec.cpp:10

PDE::NoFluxBoundaries

void NoFluxBoundaries(void)

Implementation of no-flux boundaries.

**Definition:** pde.cpp:248

PDE::Plot

void Plot(Graphics \*g, const int layer=0)

Plots one layer of the PDE plane to a Graphics window.

**Definition:** pde.cpp:111

PDE::SizeX

int SizeX() const

Returns the horizontal size of the PDE planes.

**Definition:** pde.h:74

PDE::~PDE

virtual ~PDE()

**Definition:** pde.cpp:64

PDE::layers

int layers

**Definition:** pde.h:240

PDE::PDE

PDE(void)

empty constructor (necessary for derivation)

**Definition:** pde.cpp:54

PDE::AllocateSigma

virtual double \*\*\* AllocateSigma(const int layers, const int sx, const int sy)

Allocates a PDE plane (internal use).

**Definition:** pde.cpp:79

PDE::sizex

int sizex

**Definition:** pde.h:238

PDE::sizey

int sizey

**Definition:** pde.h:239

CellularPotts

**Definition:** ca.h:57

PDE::Max

double Max(int l)

Gets the maximum value of PDE layer l.

**Definition:** pde.h:125

PDE::Sigma

double Sigma(const int layer, const int x, const int y) const

Returns the value of grid point x,y of PDE plane "layer".

**Definition:** pde.h:95

PDE::PeriodicBoundaries

void PeriodicBoundaries(void)

Implementation of periodic boundaries.

**Definition:** pde.cpp:288

PDE

**Definition:** pde.h:31

PDE::MapColour

virtual int MapColour(double val)

Used in Plot. Takes a color and turns it into a grey value.

PDE::SizeY

int SizeY() const

Returns the vertical size of the PDE planes.

**Definition:** pde.h:79

PDE::GradC

void GradC(int layer=0, int first\_grad\_layer=1)

**Definition:** pde.cpp:304

PDE::PlotVectorField

void PlotVectorField(Graphics &g, int stride, int linelength, int first\_grad\_layer=1)

**Definition:** pde.cpp:341

PDE::GetChemAmount

double GetChemAmount(const int layer=-1)

Returns summed amount of chemical in PDE plane "layer".

**Definition:** pde.cpp:221

graph.h

PDE::addtoValue

void addtoValue(const int layer, const int x, const int y, const double value)

Adds a number to a PDE grid point.

**Definition:** pde.h:116

PDE::Min

double Min(int l)

Returns the minimum value of PDE layer l.

**Definition:** pde.h:139

Graphics

API for Graphics windows.

**Definition:** graph.h:36

PDE::AbsorbingBoundaries

void AbsorbingBoundaries(void)

Implementation of absorbing boundaries.

**Definition:** pde.cpp:270

PDE::ContourPlot

void ContourPlot(Graphics \*g, int layer=0, int colour=1)

Plots the PDE field using contour lines.

**Definition:** pde.cpp:142

PDE::setValue

void setValue(const int layer, const int x, const int y, const double value)

Sets grid point x,y of PDE plane "layer" to value "value".

**Definition:** pde.h:106

PDE::Layers

int Layers() const

Returns the number of PDE layers in the PDE object.

**Definition:** pde.h:84

PDE::sigma

double \*\*\* sigma

**Definition:** pde.h:229

PDE::Diffuse

void Diffuse(int repeat)

Carry out $n$ diffusion steps for all PDE planes.

**Definition:** pde.cpp:178

min

#define min(x, y)

**Definition:** conrec.cpp:9

PDE::Secrete

void Secrete(CellularPotts \*cpm)

Reaction and interaction of CPM plane with PDE planes.

Info

Enables interactive querying of the simulation.

**Definition:** info.h:36


---

Generated on Thu Aug 14 2014 22:04:01 for Tissue Simulation Toolkit by  

 1.8.6
