# Supplementary material for: Crawling and Gliding: A Computational Model for Shape-Driven Cell Migration
Source: PLoS Comput Biol. 2015 Oct 21;11(10):e1004280. doi: 10.1371/journal.pcbi.1004280 (PMC4619082; doi:10.1371/journal.pcbi.1004280)
Supplement: S1 Code — (ZIP) [file pcbi.1004280.s012.zip › release/tst/doc/html/annotated.html]

Tissue Simulation Toolkit: Class List


|  |
| --- |
| Tissue Simulation Toolkit  0.1.4.1 |


- Main Page
- Namespaces
- Classes
- Files

- Class List
- Class Hierarchy
- Class Members

Class List

Here are the classes, structs, unions and interfaces with brief descriptions:

|  |  |
| --- | --- |
| ColourMode |  |
| Cell |  |
| CellularPotts |  |
| co |  |
| Dir |  |
| Dish | The virtual Petri dish |
| Graphics | API for Graphics windows |
| Info | Enables interactive querying of the simulation |
| li |  |
| Parameter |  |
| PDE |  |
| Point |  |
| QtGraphics |  |
| X11Graphics | X-Windows implementation of Graphics interface |


---

Generated on Thu Aug 14 2014 22:04:01 for Tissue Simulation Toolkit by  

 1.8.6
