# Supplementary material for: Crawling and Gliding: A Computational Model for Shape-Driven Cell Migration
Source: PLoS Comput Biol. 2015 Oct 21;11(10):e1004280. doi: 10.1371/journal.pcbi.1004280 (PMC4619082; doi:10.1371/journal.pcbi.1004280)
Supplement: S1 Code — (ZIP) [file pcbi.1004280.s012.zip › release/tst/doc/html/classPDE-members.html]

Tissue Simulation Toolkit: Member List


|  |
| --- |
| Tissue Simulation Toolkit  0.1.4.1 |


- Main Page
- Namespaces
- Classes
- Files

- Class List
- Class Hierarchy
- Class Members

PDE Member List

This is the complete list of members for PDE, including all inherited members.

|  |  |  |
| --- | --- | --- |
| AbsorbingBoundaries(void) | PDE |  |
| addtoValue(const int layer, const int x, const int y, const double value) | PDE | inline |
| AllocateSigma(const int layers, const int sx, const int sy) | PDE | protectedvirtual |
| alt\_sigma | PDE | protected |
| ContourPlot(Graphics \*g, int layer=0, int colour=1) | PDE |  |
| Diffuse(int repeat) | PDE |  |
| GetChemAmount(const int layer=-1) | PDE |  |
| GradC(int layer=0, int first\_grad\_layer=1) | PDE |  |
| Info class | PDE | friend |
| Layers() const | PDE | inline |
| layers | PDE | protected |
| MapColour(double val) | PDE | protectedvirtual |
| Max(int l) | PDE | inline |
| Min(int l) | PDE | inline |
| NoFluxBoundaries(void) | PDE |  |
| PDE(const int layers, const int sizex, const int sizey) | PDE |  |
| PDE(void) | PDE | protected |
| PeriodicBoundaries(void) | PDE |  |
| Plot(Graphics \*g, const int layer=0) | PDE |  |
| Plot(Graphics \*g, CellularPotts \*cpm, const int layer=0) | PDE |  |
| PlotVectorField(Graphics &g, int stride, int linelength, int first\_grad\_layer=1) | PDE |  |
| Secrete(CellularPotts \*cpm) | PDE |  |
| setValue(const int layer, const int x, const int y, const double value) | PDE | inline |
| sigma | PDE | protected |
| Sigma(const int layer, const int x, const int y) const | PDE | inline |
| SizeX() const | PDE | inline |
| sizex | PDE | protected |
| sizey | PDE | protected |
| SizeY() const | PDE | inline |
| TheTime(void) const | PDE | inline |
| ~PDE() | PDE | virtual |


---

Generated on Thu Aug 14 2014 22:04:01 for Tissue Simulation Toolkit by  

 1.8.6
