# Supplementary material for: Crawling and Gliding: A Computational Model for Shape-Driven Cell Migration
Source: PLoS Comput Biol. 2015 Oct 21;11(10):e1004280. doi: 10.1371/journal.pcbi.1004280 (PMC4619082; doi:10.1371/journal.pcbi.1004280)
Supplement: S1 Code — (ZIP) [file pcbi.1004280.s012.zip › release/tst/doc/html/parameter_8h.html]

Tissue Simulation Toolkit: parameter.h File Reference


|  |
| --- |
| Tissue Simulation Toolkit  0.1.4.1 |


- Main Page
- Namespaces
- Classes
- Files

- File List
- File Members

Classes |
Functions

parameter.h File Reference

`#include <iostream>`

Include dependency graph for parameter.h:

This graph shows which files directly or indirectly include this file:

Go to the source code of this file.

|  |  |
| --- | --- |
| Classes | |
| class | Parameter |
|  | |

|  |  |
| --- | --- |
| Functions | |
| ostream & | operator<< (ostream &os, Parameter &p) |
|  | |
| const char \* | sbool (const bool &p) |
|  | |

## Function Documentation

|  |  |  |  |
| --- | --- | --- | --- |
| ostream& operator<< | ( | ostream & | *os*, |
|  |  | Parameter & | *p* |
|  | ) |  |  |

References Parameter::Write().

|  |  |  |  |  |  |
| --- | --- | --- | --- | --- | --- |
| const char\* sbool | ( | const bool & | *p* | ) |  |

Referenced by Parameter::Write().


---

Generated on Thu Aug 14 2014 22:04:01 for Tissue Simulation Toolkit by  

 1.8.6
