# Supplementary figures and images for: Crawling and Gliding: A Computational Model for Shape-Driven Cell Migration
Source: PLoS Comput Biol. 2015 Oct 21;11(10):e1004280. doi: 10.1371/journal.pcbi.1004280 (PMC4619082; doi:10.1371/journal.pcbi.1004280)

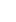

Supplement: S1 Code — (ZIP) [file pcbi.1004280.s012.zip › release/tst/doc/html/ftv2lastnode.png]

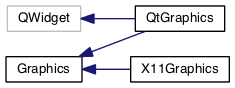

Supplement: S1 Code — (ZIP) [file pcbi.1004280.s012.zip › release/tst/doc/html/inherit_graph_10.png]

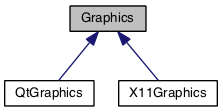

Supplement: S1 Code — (ZIP) [file pcbi.1004280.s012.zip › release/tst/doc/html/classGraphics__inherit__graph.png]

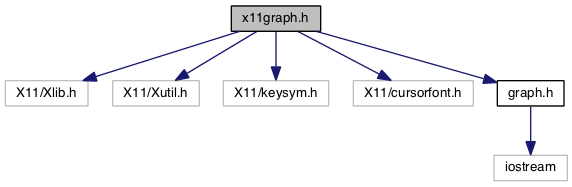

Supplement: S1 Code — (ZIP) [file pcbi.1004280.s012.zip › release/tst/doc/html/x11graph_8h__incl.png]

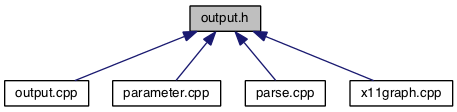

Supplement: S1 Code — (ZIP) [file pcbi.1004280.s012.zip › release/tst/doc/html/output_8h__dep__incl.png]

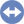

Supplement: S1 Code — (ZIP) [file pcbi.1004280.s012.zip › release/tst/doc/html/sync_on.png]

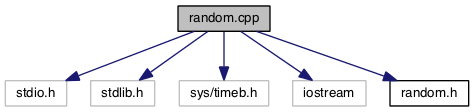

Supplement: S1 Code — (ZIP) [file pcbi.1004280.s012.zip › release/tst/doc/html/random_8cpp__incl.png]

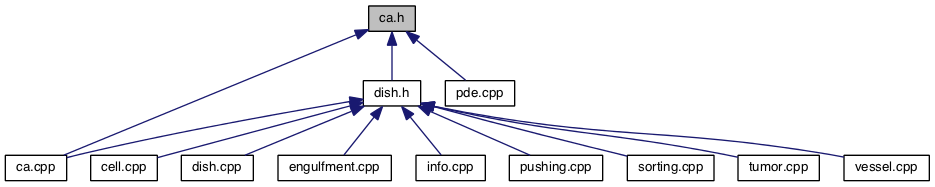

Supplement: S1 Code — (ZIP) [file pcbi.1004280.s012.zip › release/tst/doc/html/ca_8h__dep__incl.png]

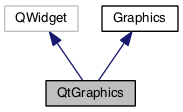

Supplement: S1 Code — (ZIP) [file pcbi.1004280.s012.zip › release/tst/doc/html/classQtGraphics__coll__graph.png]

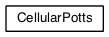

Supplement: S1 Code — (ZIP) [file pcbi.1004280.s012.zip › release/tst/doc/html/inherit_graph_1.png]

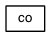

Supplement: S1 Code — (ZIP) [file pcbi.1004280.s012.zip › release/tst/doc/html/inherit_graph_2.png]

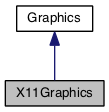

Supplement: S1 Code — (ZIP) [file pcbi.1004280.s012.zip › release/tst/doc/html/classX11Graphics__inherit__graph.png]

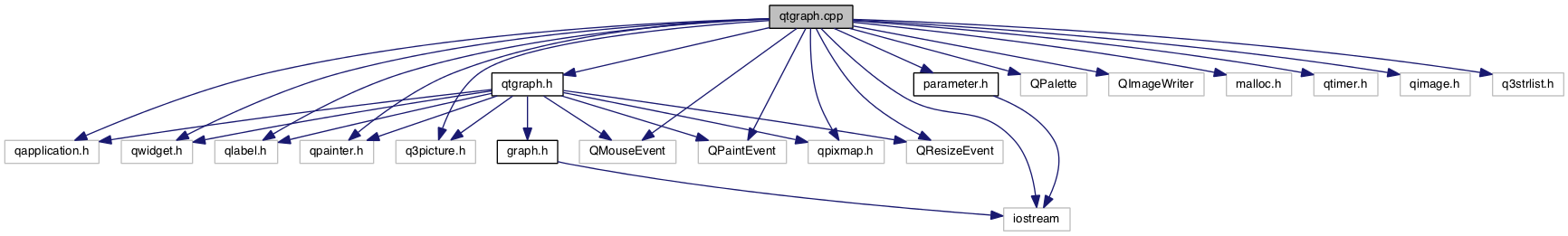

Supplement: S1 Code — (ZIP) [file pcbi.1004280.s012.zip › release/tst/doc/html/qtgraph_8cpp__incl.png]

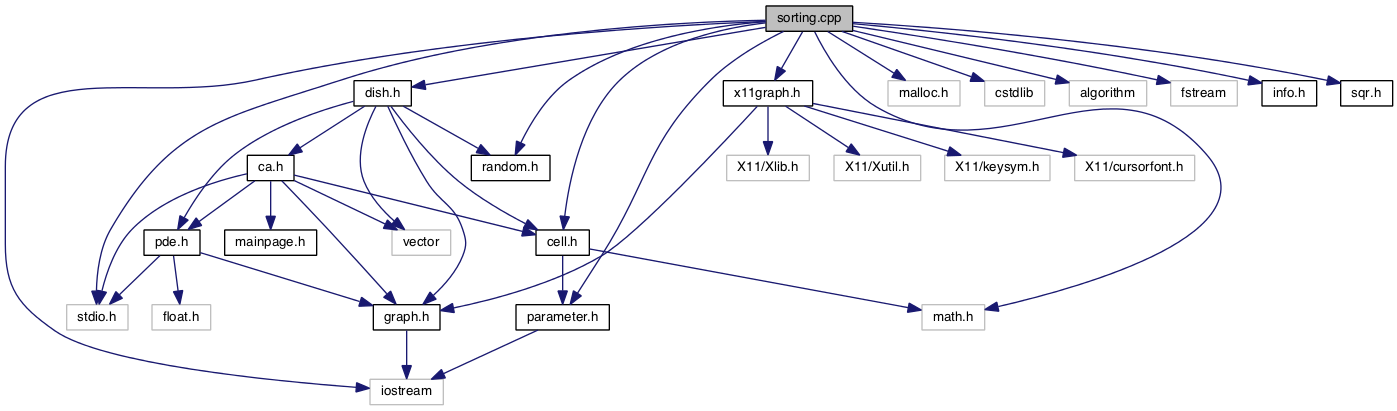

Supplement: S1 Code — (ZIP) [file pcbi.1004280.s012.zip › release/tst/doc/html/sorting_8cpp__incl.png]

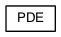

Supplement: S1 Code — (ZIP) [file pcbi.1004280.s012.zip › release/tst/doc/html/inherit_graph_8.png]

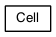

Supplement: S1 Code — (ZIP) [file pcbi.1004280.s012.zip › release/tst/doc/html/inherit_graph_0.png]

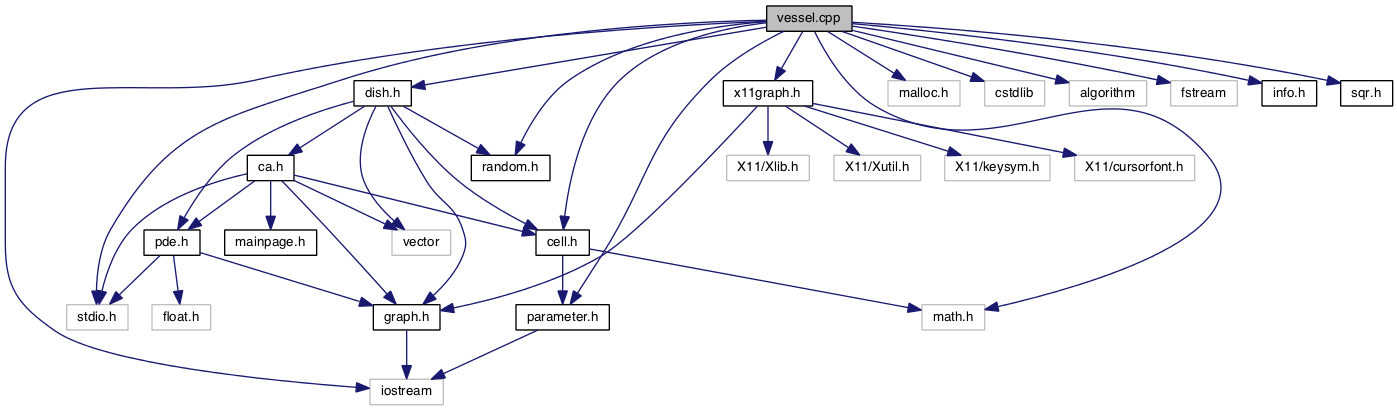

Supplement: S1 Code — (ZIP) [file pcbi.1004280.s012.zip › release/tst/doc/html/vessel_8cpp__incl.png]

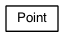

Supplement: S1 Code — (ZIP) [file pcbi.1004280.s012.zip › release/tst/doc/html/inherit_graph_9.png]

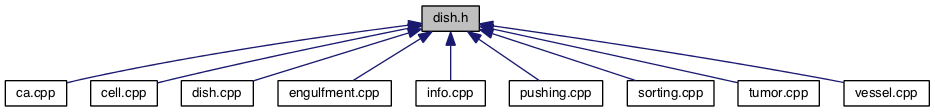

Supplement: S1 Code — (ZIP) [file pcbi.1004280.s012.zip › release/tst/doc/html/dish_8h__dep__incl.png]

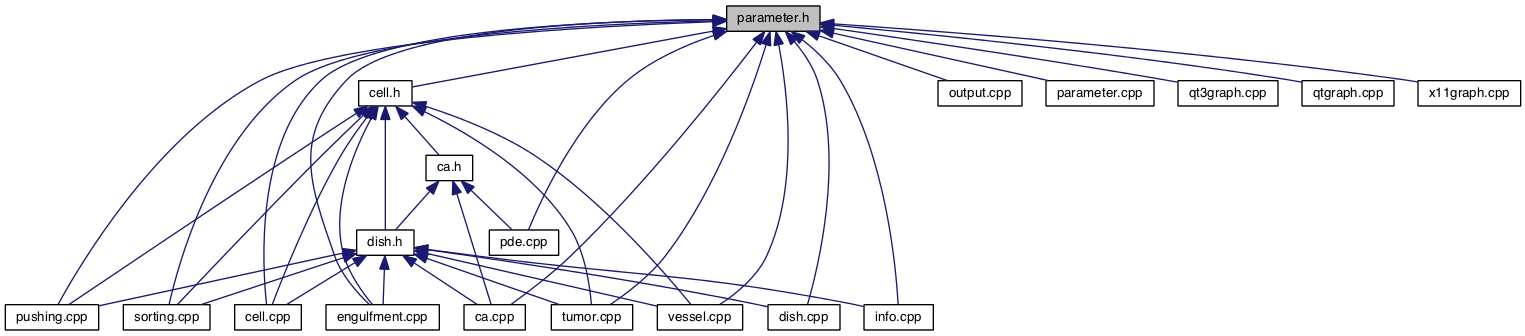

Supplement: S1 Code — (ZIP) [file pcbi.1004280.s012.zip › release/tst/doc/html/parameter_8h__dep__incl.png]

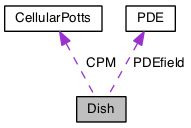

Supplement: S1 Code — (ZIP) [file pcbi.1004280.s012.zip › release/tst/doc/html/classDish__coll__graph.png]

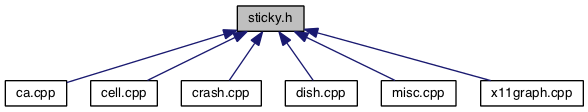

Supplement: S1 Code — (ZIP) [file pcbi.1004280.s012.zip › release/tst/doc/html/sticky_8h__dep__incl.png]

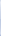

Supplement: S1 Code — (ZIP) [file pcbi.1004280.s012.zip › release/tst/doc/html/tab_b.png]

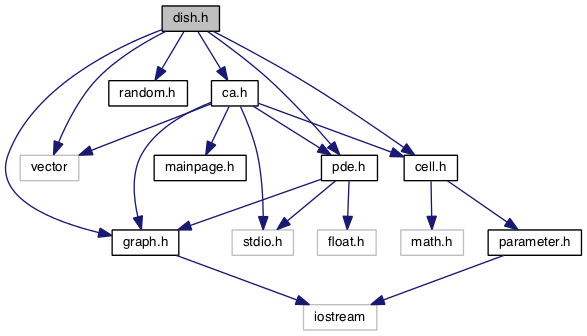

Supplement: S1 Code — (ZIP) [file pcbi.1004280.s012.zip › release/tst/doc/html/dish_8h__incl.png]

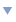

Supplement: S1 Code — (ZIP) [file pcbi.1004280.s012.zip › release/tst/doc/html/ftv2mnode.png]

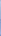

Supplement: S1 Code — (ZIP) [file pcbi.1004280.s012.zip › release/tst/doc/html/tab_h.png]
